# Guaranteed State Estimation Using a Bundle of Interval Observers with Adaptive Gains Applied to the Induction Machine

**DOI:** 10.3390/s21082584

**Published:** 2021-04-07

**Authors:** Manuel Schwartz, Stefan Krebs, Sören Hohmann

**Affiliations:** Institute of Control Systems, Karlsruhe Institute of Technology, 76131 Karlsruhe, Germany; stefan.krebs@partner.kit.edu (S.K.); soeren.hohmann@kit.edu (S.H.)

**Keywords:** interval observer, bundle of observers, reduced-order hybrid observer, adaptive observer, induction machine

## Abstract

The scope of this paper is the design of an interval observer bundle for the guaranteed state estimation of an uncertain induction machine with linear, time-varying dynamics. These guarantees are of particular interest in the case of safety-critical systems. In many cases, interval observers provide large intervals for which the usability becomes impractical. Hence, based on a reduced-order hybrid interval observer structure, the guaranteed enclosure within intervals of the magnetizing current’s estimates is improved using a bundle of interval observers. One advantage of such an interval observer bundle is the possibility to reinitialize the interval observers at specified timesteps during runtime with smaller initial intervals, based on previously observed system states, resulting in decreasing interval widths. Thus, unstable observer dynamics are considered so as to take advantage of their transient behavior, whereby the overall stability of the interval estimation is maintained. An algorithm is presented to determine the parametrization of reduced-order interval observers. To this, an adaptive observer gain is introduced with which the system states are observed optimally by considering a minimal interval width at variable operating points. Furthermore, real-time capability and validation of the proposed methods are shown. The results are discussed with simulations as well as experimental data obtained with a test bench.

## 1. Motivation and Overview

Guaranteed state estimation of uncertain linear continuous time dynamical systems with time-varying parameters is of increasing interest in various technical applications. Firstly presented for the induction machine in [1], a reduced-order hybrid interval observer for the verified state estimation was designed. Its main advantage is the provision of guarantees regarding the estimated states. A major disadvantage is the relatively wide interval width resulting in practical inapplicability. To tackle this challenge, a bundle of interval observers with optimal adaptive gains regarding the operation point of the plant is designed within this paper leading to a significant reduction of the estimated interval widths.

Challenges for guaranteed state estimation are the growing complexity of technical systems in combination with a great demand for functional safety, e.g., autonomous driving [2], which results in requirements for fault detection and fault diagnosis applications guaranteeing the safe and reliable operation of the system [3,4]. Furthermore, in the presence of undesirable effects such as failures in the actuators or sensors, it is desired that the control system is responsive and adaptive to such failures and adjustable to recover the system from anomalies and failures [5]. Therefore, the aim of a diagnosis system is to detect faults as early as possible while, at the same time, false alarms, e.g., due to measurement noise or parameter uncertainties exceeding acceptable bounds, have to be avoided to minimize unnecessary system shutdowns and maximize system reliability and dependability [6]. For these mentioned challenges and applications, guaranteed state estimation using real-time capable interval observers are an appropriate choice. An overview about interval estimation applied to the diagnosis and control of uncertain systems is given in Raissi et al. [7].

One way to achieve guaranteed enclosure is the observation of system states by considering input and output noise as well as parameter uncertainties in a model-based estimation setup resulting in essential signals for fault diagnosis and other applications. Under certain conditions and restrictions, the estimation of the important system states gives guaranteed results leading to safety diagnosis based on guaranteed intervals for the signals instead of a measurement value trend or threshold comparison [8].

However, approaches like the extended Luenberger Observer, the extended Kalman Filter, the High-Gain Observer, the Sliding Mode Observer, the $H_{\infty }$ Observer, or the $L_{2}$-Gain Observer are not directly applicable with a priori assumptions on probability distributions or models of the uncertainties, because these are often unknown for practical applications. Furthermore, such methods, e.g., Sun et al. [9], cannot be used because they do not provide necessary guaranteed error bounds [10]. Therefore, it is reasonable to utilize methods that assume that the uncertainties are unknown but their boundaries are specified, which is usually given for practical applications.

In the literature, there are two approaches which should be emphasized considering bounded intervals for the uncertainties, namely set-membership estimators and interval observers [11,12]. Both aim at providing sets or intervals containing all the possible state vectors consistent with the model structure, and input and output uncertainties as well as uncertainties in initial states and the parameters.

Set-membership estimators are based on a predictor-corrector structure with different descriptions of uncertainties, e.g., zonotopes, parallelotopes, subpavings, and ellipsoids [6,13]. These approaches have issues with real-time capability due to excessive computational effort for the complex and guaranteed description of the uncertainties. Hence, these methods are not suitable for systems with relatively small time constants like the induction machine. However, in Zbranek and Vesely [14], a set-membership estimator for a permanent magnet synchronous machine based on a nonlinear model with four states is designed. The results show that this approach is only suitable for online operations under restrictive conditions. In the case of a induction machine with similar time constants, the coupling of such state estimators to a bundle to improve the performance is not feasible.

In contrast, interval observers are based on the classical observer structure. For each state a lower and an upper bound is estimated. Due to a less complex description of the set of possible states, i.e., just a box, the computational effort is lower than with the aforementioned approach. This method is divided into cooperative-based and hybrid interval observers [12].

The idea behind the so-called hybrid interval observer is to design dynamical systems with monotone error dynamics that are activated on certain switching conditions guarding the states which change the monotonicity of the system [10,15]. These systems enclose all the state trajectories, generated by the original uncertain system, by estimating a lower and an upper boundary for each state in a guaranteed way [16,17]. The derivatives of the error dynamics activate a subsequent system, keeping monotonicity of the error dynamics [18]. Finally, switching conditions describe the change of these derivatives. This leads to a cooperative system [19] for the error dynamics, resulting in an inclusion of the real state.

Both approaches, set-membership estimators and hybrid interval observers, face the key challenge of finding a suitable parametrization for the observer or the estimator in order to get a sufficient tight interval for the estimated states. With the task of considering input and output noise as well as parameter uncertainties, these methods tend to give conservative intervals widths. Especially for electric machines whereby the system dynamics have time-varying parameters, thus the parametrization of the observer is a crucial task.

Due to the basic idea of a guaranteed enclosure of states, every interval observer limits the range of the actual value. Therefore, the actual value has to be below each upper bound and above each lower bound of the considered interval observers. Due to the fact that interval observers have to bound the same value, even with different parameterizations, these interval observers allow a comparison among themselves regarding the narrowest enclosure of the actual value for the current operating point. This allows that the envelope to be composed by the lowest upper bound and the highest lower bound chosen out of each interval observer considered in the estimation setup.

Following this idea, a so-called bundle of interval observers, for which several observers are run in parallel and the resulting interval is arranged with the most suitable upper and lower boundary, can be developed. Furthermore, within a bundle, it is possible to generate interval observers with unstable dynamics, called framers [20], which simply bound the estimated state without the assurance of stability. Finally, a better convergence rate as well as smaller interval widths are expected. Interval observer bundles, predominantly applied to bioprocessess, are presented in [21,22,23]. Furthermore, a similar approach is named a bank of observers. Hereby, the main difference between a bundle of observers and a bank of observers is the used methodical approach for a single observer as well as the underlying system description. If estimation with zonotopes is considered, the resulting guaranteed enclosure is the intersection of the solutions [24]. Hence, this would be the set-membership approach pendant to the bundle approach presented in this paper. Secondly, if uncertain switching functions are considered to maintain guaranteed interval enclosure for nonlinear systems, the so-called global solution would be the over-approximation of activated interval observers [25]. This stabilizes the guaranteed boundaries but does not minimize the error bounds.

However, most publications on interval observers and bundle of observers do neither investigate the selection of observer gains, the behavior of unstable observer dynamics, or an algorithm to determine feasible configurations with respect to an improvement of the interval width. Their focus is on the design for a suitable structure of a single interval observer. This strongly depends on the underlying model, hence, this usually is a challenging task. A subsequent arrangement to a bundle of observers is made using an arbitrary or a not precisely specified parametrization of the observer gain, which is not discussed in detail.

In this contribution, to tackle the gap between using a bundle of interval observers to improve state estimation and obtain an optimal solution for the given task, scenario-based optimization for the reduced-order hybrid interval observer’s parametrization for a linear time-varying model of the induction machine is discussed. Hereby, unstable observer dynamics are considered. Moreover, the results of an offline optimization are used online to adapt the observer gains during runtime.

Within previously published guaranteed interval estimation techniques [1,10,15,26,27], realistic sensor errors have not been considered and interval widths need to be minimized further in order to be applied for a practical algorithm (e.g., fault detection). For such practical usage of guaranteed interval estimation, the interval width needs to be as small as possible. Hence, to improve the interval widths for guaranteed state estimation with a bundle of observers, the reduced-order interval observer approach has been chosen. However, all these approaches are capable of being used in an observer bundle as presented within this contribution.

This paper is organized as follows: First, some necessary interval arithmetics, coordinate transformations, and matrix operations are given. Afterwards, the design of the reduced-order hybrid interval observer is presented. A description of an interval observer bundle using some preliminary work out of Moisan et al. [23] as well as the necessary definitions for the hybrid interval observer and the re-initialization approach with an overview of some re-initialization conditions are introduced. In Section 3, a model of the induction machine in αβ-coordinates is presented. The Section 4 deals with the design and implementation of the reduced-order hybrid interval observer and the combination to the interval observer bundle. A general time-invariant as well as a time-varying observer gain are specified. Furthermore, an algorithm for the parametrization of the observer bundle as well as the reduced-order interval observers with a scenario-based optimization is also presented in the Section 4. In the process, resulting lookup tables and a switching algorithm suitable for the adaptive gain are presented.

The validation of the interval observer bundle is executed by comparing the measured torque with the one calculated with the stator currents under consideration of the respective uncertainties and the estimated magnetizing current intervals. This is reasonable since the measurement of the magnetic flux is usually a sophisticated and expensive task.

Finally, the implementation of the whole system along with the re-initialization process is discussed for the induction machine with simulations and measurement data in Section 5.

## 2. Preliminaries and Methods

This section presents necessary coordinate transformations, interval arithmetics, and methods in order to establish the reduced-order hybrid interval observer and the bundle of interval observers.

### 2.1. Clarke Transformation

With this transformation, the states of an induction machine within the three phase system $z_{v}\left(t\right)$ are transformed into the αβ-coordinate system $z_{q}\left(t\right)$. To be able to consider uncertain but bounded states of the three-phase system $z_{v}\left(t\right)$ with v∈[1,2,3] represented by intervals $\left[\;{\underline{z}}_{v}\left(t\right),{\bar{z}}_{v}\left(t\right)\right]$, Definition 1 is used. The interval transformation (3) is obtained by applying basic interval arithmetic operations as defined in Jaulin et al. [13].

**Definition** **1.** *[10] An interval Clarke transformation guaranteeing:*

(1) $${\underline{z}}_{q}\left(t\right)\leq z_{q}\left(t\right)\leq {\bar{z}}_{q}\left(t\right),\;q\in \left\{\alpha ,\beta \right\}$$

*under consideration of uncertain but bounded variables:*

(2) $${\underline{z}}_{v}\left(t\right)\leq z_{v}\left(t\right)\leq {\bar{z}}_{v}\left(t\right),\;v\in \left\{1,2,3\right\}$$

*is given by:*

(3) $$\left[\begin{array}{c} {\bar{z}}_{\alpha }\left(t\right)\\ {\bar{z}}_{\beta }\left(t\right)\\ {\underline{z}}_{\alpha }\left(t\right)\\ {\underline{z}}_{\beta }\left(t\right) \end{array}\right]=\frac{2}{3}\;\cdot \;\left[\begin{array}{cccccc} 1 & 0 & 0 & 0 & -\frac{1}{2} & -\frac{1}{2}\\ 0 & \frac{\sqrt{3}}{2} & 0 & 0 & 0 & -\frac{\sqrt{3}}{2}\\ 0 & -\frac{1}{2} & -\frac{1}{2} & 1 & 0 & 0\\ 0 & 0 & -\frac{\sqrt{3}}{2} & 0 & \frac{\sqrt{3}}{2} & 0 \end{array}\right]\;\cdot \;\left[\begin{array}{c} {\bar{z}}_{1}\left(t\right)\\ {\bar{z}}_{2}\left(t\right)\\ {\bar{z}}_{3}\left(t\right)\\ {\underline{z}}_{1}\left(t\right)\\ {\underline{z}}_{2}\left(t\right)\\ {\underline{z}}_{3}\left(t\right) \end{array}\right].$$

### 2.2. Matrix Operations

In order to provide a compact description of the interval observer, we define some matrix operations in Definition 2. Hereby, Equations (4) and (5) provide a Metzler matrix $\tilde{W}$ with the main diagonal elements $w_{ij}$ with i=j and the absolute values $\left|w_{ij}\right|$ on the secondary diagonals with i≠j of the original matrix W. Furthermore, ${\tilde{P}}^{+}$ is obtained considering only positive values and ${\tilde{P}}^{-}$ only negative values of a Metzler matrix. Finally, the matrices $M^{+}$ and $M^{-}$ are given with the operations (10) and (11) necessary for the interval state space representation of a dynamic system.

**Definition** **2.** (4) $$\tilde{W}=\left({\tilde{w}}_{ij}\right)=:M\left\{W=\left(w_{ij}\right)\right\}$$

(5) $$\begin{array}{cc} {\tilde{w}}_{ij}= & \left\{\begin{array}{cc} w_{ij} & ,if\;\left(i=j\right)\\ \left|w_{ij}\right| & ,\mathrm{otherwise} \end{array}\right. \end{array}$$

(6) $${\tilde{P}}^{+}=\left({\tilde{p}}_{ij}^{+}\right)=:{\tilde{P}}^{+}\left\{P=\left(p_{ij}\right)\right\}$$

(7) $$\begin{array}{cc} {\tilde{p}}_{ij}^{+}= & \left\{\begin{array}{cc} p_{ij} & ,if\;\left(i=j\wedge p_{ij}\geq 0\right)\\ 0 & ,\mathrm{otherwise} \end{array}\right. \end{array}$$

(8) $${\tilde{P}}^{-}=\left({\tilde{p}}_{ij}^{-}\right)=:{\tilde{P}}^{-}\left\{P=\left(p_{ij}\right)\right\}$$

(9) $$\begin{array}{cc} {\tilde{p}}_{ij}^{-}= & \left\{\begin{array}{cc} p_{ij} & ,if\;\left(i\neq j\wedge p_{ij}<0\right)\\ 0 & ,\mathrm{otherwise} \end{array}\right. \end{array}$$

(10) $$M^{+}=\max\left(0,M\right)=:P^{+}\left\{M\right\}$$

(11) $$M^{-}=\min\left(0,M\right)=:P^{-}\left\{M\right\}=M-M^{+}$$

### 2.3. Reduced-Order Interval Observer

Subsequently, some preliminaries for the design of a reduced-order interval observer are proposed.

**Lemma** **1.** *According to Angeli and Sontag [28], a system:*

(12) $$\dot{x}\left(t\right)=A\left(x,t\right)\;\cdot \;x\left(t\right)+b\left(x,t\right)$$

*is called positive if $A\left(x,t\right)$ is Metzler and $b\left(x,t\right)$ is nonnegative, hence $x\left(t\right)$ is nonnegative for all t if $x\left(t_{0}\right)$ is nonnegative.*

Hereby, the system can be nonlinear. However, the same properties hold for a time-varying linear system:

(13) $$\begin{array}{c} \begin{array}{cc} \dot{x}\left(t\right)\; & =A\left(t\right)\;\cdot \;x\left(t\right)+B\left(t\right)\;\cdot \;u\left(t\right)\\ y\left(t\right)\; & =C\left(t\right)\;\cdot \;x\left(t\right) \end{array}. \end{array}$$

**Assumption** **1.** *For all t, there exists a one times differentiable $L\left(t\right)$ such that:*

(14) $$\tilde{F}=M\left\{F\right\}$$

*with:*

(15) $$F=A_{22}\left(t\right)-L\left(t\right)\;\cdot \;A_{12}\left(t\right)$$

*is time-invariant and Hurwitz.*

*Herein, $A_{12}\left(t\right)\in R^{q\times \left(n-q\right)}$ and $A_{22}\left(t\right)\in R^{\left(n-q\right)\times \left(n-q\right)}$ are elements of the block matrix $A\left(t\right)\in R^{n\times n}$ given as:*

(16) $$A\left(t\right)=\left[\begin{array}{cc} A_{11}\left(t\right) & A_{12}\left(t\right)\\ A_{21}\left(t\right) & A_{22}\left(t\right) \end{array}\right].$$

A Hurwitz matrix is a structured real square matrix constructed with coefficients of a real polynomial, whose zeros lie in the closed left half-plane of the complex plane, yielding a totally nonnegative matrix. It should be noted that the design of $L\left(t\right)$ has to be done in order to meet the requirements of Assumption 1.

For the reduced-order Luenberger Observer, the state vector is divided into measurable states $y\left(t\right)\in R^{q}$ and the unmeasurable states $r\left(t\right)\in R^{n-q}$ with:

(17) $$\begin{array}{c} x\left(t\right)=\left[\begin{array}{c} y(t)\\ r(t) \end{array}\right]. \end{array}$$

For the following Proposition 1, matrix operations (4) to (11) are applied.

**Proposition** **1.** *If Assumption 1 holds, then a reduced-order interval observer estimating $\left[r\left(t\right)\right]$ with $u\left(t\right)=\left[\underline{u},\bar{u}\right]$ and $y\left(t\right)=\left[\underline{y},\bar{y}\right]$ is given by:*

(18) $$\begin{array}{cc} \rho \left(t\right) & =r\left(t\right)-L\left(t\right)\;\cdot \;y\left(t\right), \end{array}$$

(19) $$\begin{array}{cc} \;x\left(t_{0}\right) & \in \left[\underline{x}\left(t_{0}\right),\bar{x}\left(t_{0}\right)\right],\; \end{array}$$

(20) $$\begin{array}{cc} \left[\begin{array}{c} \bar{\rho }\left(t_{0}\right)\\ \underline{\rho }\left(t_{0}\right)\; \end{array}\right]= & \left[\begin{array}{c} \bar{r}\left(t_{0}\right)\\ \underline{r}\left(t_{0}\right)\; \end{array}\right]-\left[\begin{array}{cc} L^{+}\left(t_{0}\right) & L^{-}\left(t_{0}\right)\\ L^{-}\left(t_{0}\right) & L^{+}\left(t_{0}\right) \end{array}\right]\;\cdot \;\left[\begin{array}{c} \bar{y}\left(t_{0}\right)\\ \underline{y}\left(t_{0}\right) \end{array}\right]. \end{array}$$

*Hereby, $\rho \left(t\right)$ are the states of the reduced-order interval observer, obtained with transformation *(18)*. Equation *(20)* provides the initial states. The dynamics of the reduced-order interval observer is given as:*

(21) $$\begin{array}{cc} \left[\begin{array}{c} \dot{\bar{\rho }}\left(t\right)\\ \dot{\underline{\rho }}\left(t\right)\; \end{array}\right]= & \overset{F}{\overset{︷}{\left[\begin{array}{cc} {\tilde{F}}^{+} & {\tilde{F}}^{-}\\ {\tilde{F}}^{-} & {\tilde{F}}^{+} \end{array}\right]}}\;\cdot \;\left[\begin{array}{c} \bar{\rho }\left(t\right)\\ \underline{\rho }\left(t\right)\; \end{array}\right]+\overset{G\left(t\right)}{\overset{︷}{\left[\begin{array}{cc} G^{+}\left(t\right) & G^{-}\left(t\right)\\ G^{-}\left(t\right) & G^{+}\left(t\right) \end{array}\right]}}\;\cdot \;\left[\begin{array}{c} \bar{y}\left(t\right)\\ \underline{y}\left(t\right) \end{array}\right]+\overset{H\left(t\right)}{\overset{︷}{\left[\begin{array}{cc} H^{+}\left(t\right) & H^{-}\left(t\right)\\ H^{-}\left(t\right) & H^{+}\left(t\right) \end{array}\right]}}\;\cdot \;\left[\begin{array}{c} \bar{u}\left(t\right)\\ \underline{u}\left(t\right) \end{array}\right], \end{array}$$

*with the matrices based on the time-varying system description (13):*

(22) $$B\left(t\right)=\left[\begin{array}{c} B_{1}\left(t\right)\in R^{q\times p}\\ B_{2}\left(t\right)\in R^{\left(n-q\right)\times p} \end{array}\right],$$

(23) $$H\left(t\right)=B_{2}\left(t\right)-L\left(t\right)\;\cdot \;B_{1}\left(t\right),$$

(24) $$\begin{array}{cc} G(t)= & \left(A_{22}\left(t\right)-L\left(t\right)\;\cdot \;A_{12}\left(t\right)\right)\;\cdot \;L\left(t\right)\\ \; & +A_{21}\left(t\right)-L\left(t\right)\;\cdot \;A_{11}\left(t\right)-\dot{L}\left(t\right) \end{array}$$

*and the construction as Metzler matrices:*

(25a) $$\begin{array}{cccc} & {\tilde{F}}^{+}={\tilde{P}}^{+}\left\{F\right\},\; & & {\tilde{F}}^{-}={\tilde{P}}^{-}\left\{F\right\}, \end{array}$$

(25b) $$\begin{array}{cccc} & G^{+}\left(t\right)=P^{+}\left\{G\left(t\right)\right\},\; & & G^{-}\left(t\right)=P^{-}\left\{G\left(t\right)\right\}, \end{array}$$

(25c) $$\begin{array}{cccc} & H^{+}\left(t\right)=P^{+}\left\{H\left(t\right)\right\},\; & & H^{-}\left(t\right)=P^{-}\left\{H\left(t\right)\right\}, \end{array}$$

*as well as the inverse interval transformation leading the states to be estimated:*

(26) $$\begin{array}{cc} \left[\begin{array}{c} \bar{r}\left(t\right)\\ \underline{r}\left(t\right)\; \end{array}\right]= & \left[\begin{array}{c} \bar{\rho }\left(t\right)\\ \underline{\rho }\left(t\right)\; \end{array}\right]+\overset{L\left(t\right)}{\overset{︷}{\left[\begin{array}{cc} L^{+}\left(t\right) & L^{-}\left(t\right)\\ L^{-}\left(t\right) & L^{+}\left(t\right) \end{array}\right]}}\;\cdot \;\left[\begin{array}{c} \bar{y}\left(t\right)\\ \underline{y}\left(t\right)\; \end{array}\right], \end{array}$$

*with $L\left(t\right)$ being chosen such that Assumption 1 is fullfilled.*

**Proof** **of** **Proposition** **1.** Initially, it is proven that the dynamical system introduced in Proposition 1 is a framer (see Remark 1) for $\rho \left(t\right)$ and afterwards, the interval width is proven to be bounded resulting in an interval observer. To prove the inclusion of $\rho \left(t\right)$, the error dynamics of $\bar{\rho }\left(t\right)$ and $\underline{\rho }\left(t\right)$ as defined in:

(27) $$\begin{array}{cc} \; & \dot{\bar{e}}\left(t\right)=\dot{\bar{\rho }}\left(t\right)-\dot{\rho }\left(t\right), \end{array}$$

(28) $$\begin{array}{cc} \; & \dot{\underline{e}}\left(t\right)=\dot{\rho }\left(t\right)-\dot{\underline{\rho }}\left(t\right) \end{array}$$

are proven to be positive systems. Under consideration of (18), (21), and (28), one gets:

(29) $$\begin{array}{cc} \overset{{\dot{e}}_{\rho }}{\overset{︷}{\left[\begin{array}{c} \dot{\bar{e}}\left(t\right)\\ \dot{\underline{e}}\left(t\right)\; \end{array}\right]}}= & \overset{F}{\overset{︷}{\left[\begin{array}{cc} {\tilde{F}}^{+} & -{\tilde{F}}^{-}\\ -{\tilde{F}}^{-} & {\tilde{F}}^{+} \end{array}\right]}}\;\cdot \;\overset{e_{\rho }\left(t\right)}{\overset{︷}{\left[\begin{array}{c} \bar{e}\left(t\right)\\ \underline{e}\left(t\right)\; \end{array}\right]}}+\overset{G\left(t\right)}{\overset{︷}{\left[\begin{array}{cc} G^{+}\left(t\right) & -G^{-}\left(t\right)\\ -G^{-}\left(t\right) & G^{+}\left(t\right) \end{array}\right]}}\;\cdot \;\overset{Y\left(t\right)}{\overset{︷}{\left[\begin{array}{c} \bar{y}\left(t\right)-y\left(t\right)\\ y\left(t\right)-\underline{y}\left(t\right)\; \end{array}\right]}}\\ \; & +\overset{H\left(t\right)}{\overset{︷}{\left[\begin{array}{cc} H^{+}\left(t\right) & -H^{-}\left(t\right)\\ -H^{-}\left(t\right) & H^{+}\left(t\right) \end{array}\right]}}\;\cdot \;\overset{U\left(t\right)}{\overset{︷}{\left[\begin{array}{c} \bar{u}\left(t\right)-u\left(t\right)\\ u\left(t\right)-\underline{u}\left(t\right)\; \end{array}\right]}}. \end{array}$$

Due to the fact that F is Metzler by construction and $G\left(t\right)$, $Y\left(t\right)$, $H\left(t\right)$, and $U\left(t\right)$ are nonnegative by construction, (29) is a positive system. To get the inverse transformation under consideration of $\left[\rho \left(t\right)\right]$ and $\left[y\left(t\right)\right]$, Equation (18) is adapted by using standard interval arithmetic operations yielding (26).

In order to prove the boundedness of the resulting estimates, the dynamics of the interval width:

(30) $$\bar{\rho }\left(t\right)-\underline{\rho }\left(t\right)=\bar{e}\left(t\right)+\underline{e}\left(t\right)$$

is proven to be stable. Its dynamics is given by:

(31) $$\begin{array}{cc} \dot{\bar{e}}\left(t\right)+\dot{\underline{e}}\left(t\right)= & \dot{\bar{\rho }}\left(t\right)-\dot{\underline{\rho }}\left(t\right)\\ = & \overset{\tilde{F}}{\overset{︷}{\left({\tilde{F}}^{+}-{\tilde{F}}^{-}\right)}}\;\cdot \;\left(\bar{\rho }\left(t\right)-\underline{\rho }\left(t\right)\right)+\left(G^{+}\left(t\right)-G^{-}\left(t\right)\right)\;\cdot \;\left(\bar{y}\left(t\right)-\underline{y}\left(t\right)\right)\\ \; & +\left(H^{+}\left(t\right)-H^{-}\left(t\right)\right)\;\cdot \;\left(\bar{u}\left(t\right)-\underline{u}\left(t\right)\right). \end{array}$$

Due to the fact that the system matrix in (31) is time-invariant, the system is stable if $\tilde{F}$ is Hurwitz. □

**Remark** **1.** *If Assumption 1 does not hold, (21) and (26) with an arbitrary $L\left(t\right)$ still represent a framer for $r\left(t\right)$ as shown in (29).*

**Figure 1:**
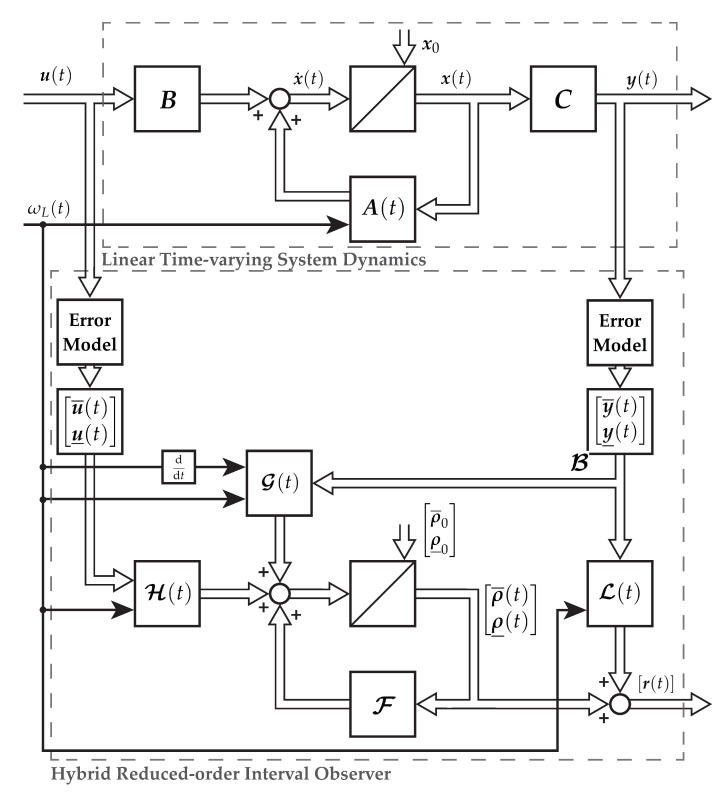
State-space representation and signal-flow of a linear time-varying system as well as the reduced-order hybrid interval observer as a block diagram.

The structure of the reduced-order interval observer is shown in Figure 1.

### 2.4. Bundle of Interval Observers

In order to improve the interval estimation for dynamic systems, a bundle of interval observers is proposed. To be consistent with the requirements and the system description as well as sensor properties, the bundle of interval observers is defined as follows.

**Definition** **3.** *An interval observer bundle 𝓑 is a finite set of b interval observers $\sum_{i}$ given as:*

(32) $$𝓑=\left\{\sum_{1},\sum_{2},\ldots ,\sum_{b}\right\},$$

*designed under the same constraints 𝓡 given as:*

(33) $$\begin{array}{c} 𝓡:\left\{\begin{array}{cc} {𝓘}_{\kappa } & =\left\{\kappa_{1},\kappa_{2},\ldots ,\kappa_{r}\right\}\\ {𝓘}_{\xi } & =\left\{\xi_{1},\xi_{2},\ldots ,\xi_{s}\right\}\\ \left(S\right) \end{array}\right., \end{array}$$

*with the number of considered uncertain parameters r∈N and uncertain output as well as input variables s∈N and the considered model of the system (S). Hereby, κ is the set of model parameters and ξ the set of output and input variables of the model.*

The definition of (S) as a determined model for the interval observer design, not necessarily done within the same approach, is reasonable in order to establish non-violating interval estimation in any case, leading to any $\sum_{i}\in 𝓑$ to be designed based on the same system model.

**Remark** **2.** *The measurement errors of the considered uncertainties ${𝓘}_{\kappa }$ and ${𝓘}_{\xi }$ for the error model are marked as follows:*

(34) $${\left\{\left[\kappa_{i}\right],\left[\xi_{j}\right]\right\}}^{\gamma \;[\mathrm{um}]\;\pm \;\eta \;\%}\;\;\mathrm{with}\;\;i=1,2,\ldots ,r\;\;\mathrm{and}\;\;j=1,2,\ldots ,s.$$

*Hereby, a bias (offset) γ coupled with the corresponding unit of measurement [um] and a relative measurement error η give further information about the underlying error model. Both values can usually be obtained by the sensor manufacturer’s information.*

To complete the description of an interval observer bundle, Definition 2 and property 3 of [23] are presented more generally. Any interval observer $\sum_{i}\in 𝓑$ encloses the actual value and therefore provides a valid upper and lower boundary. With $\underline{𝓑}\left(t\right)$ and $\bar{𝓑}\left(t\right)$ representing every upper and lower boundary produced by the interval observers within the bundle, this leads to Definition 4.

**Definition** **4.** *The best envelopes $l^{\left(𝓑\right)}\left(t\right)$ estimated by the bundle of observers 𝓑 is calculated using the equations:*

(35) $${\bar{𝓑}}_{\inf}\left(t\right)=\min\left\{\bar{𝓑}\left(t\right)\right\},$$

(36) $${\underline{𝓑}}_{\sup}\left(t\right)=\max\left\{\underline{𝓑}\left(t\right)\right\},$$

*for all $t\;\geq t_{0}$ resulting in the vector interval:*

(37) $$l^{\left(𝓑\right)}\left(t\right)=\left[{\underline{𝓑}}_{\sup}\left(t\right),\;{\bar{𝓑}}_{\inf}\left(t\right)\right].$$

Without detailed information about the properties of the interval observers, the boundedness of the bundle is given by Definition 5.

**Definition** **5.** *An interval observer bundle 𝓑, respectively the envelope $l^{\left(𝓑\right)}\left(t\right)$ is bounded, if the stability of at least one interval observer $\sum_{i}\in 𝓑$ is given.*

**Remark** **3.** *The possibility arises to supplement the bundle of observers with additional interval observers that are not necessarily developed using the same method, e.g., the interval observer proposed in [1], as long as the constraints 𝓡 given by Definition 3 are met.*

Summarized, the bundle of interval observers 𝓑 provides an interval for each estimated system state r, e.g., $\left[\begin{array}{cc} \bar{r}\left(t\right) & \underline{r}\left(t\right) \end{array}\right]$, using the reduced-order interval observer approach discussed in this paper, by any considered interval observer $\sum_{i}$ within the bundle. The tightest enclosure of the estimation $l^{\left(𝓑\right)}\left(t\right)$, given by Equations (35) and (36), leads to a single interval for each estimated system state.

### 2.5. Re-Initialization

The proposed interval observer bundle gives the possibility to use unstable framers for which the convergence of the envelope is not given, but transient behavior may improve the enclosure of the estimated values. If such unstable dynamics are used, a re-initialization procedure has to be developed.

**Definition** **6.** *Re-initialization is the procedure of restarting the integrators of $\sum_{i}$ at a specific time step $t^{R}$ with new initial values given as:*

(38) $$\left[{\underline{r}}_{0}\left(t^{R}\right),\;{\bar{r}}_{0}\left(t^{R}\right)\right]=\left[{\underline{𝓑}}_{\sup}\left(t^{R}\right),\;{\bar{𝓑}}_{\inf}\left(t^{R}\right)\right].$$

**Remark** **4.** *In case of reduced-order interval observers the initial values have to be transformed using* (20)*.*

**Remark** **5.** *The design of the bundle of interval observers and the re-initialization remain within the continuous time domain. Subsequently, the indices k and k+1 are used to support the explanations and represent two adjoining calculation steps.*

In the previous works [20,21], re-initialization is executed only at periodical time steps $t^{R}\in R_{0}^{+}$. Hereby, the best estimation performed at the end of the previous calculation period $t_{k}$ is used to reinitialize the whole bundle at $t^{R}=t_{k+1}$.

**Theorem** **1.** *To apply re-initialization for dynamic systems with relatively small time constants, re-initialization has to be performed within the same time step as measurements are obtained.*

**Figure 2:**
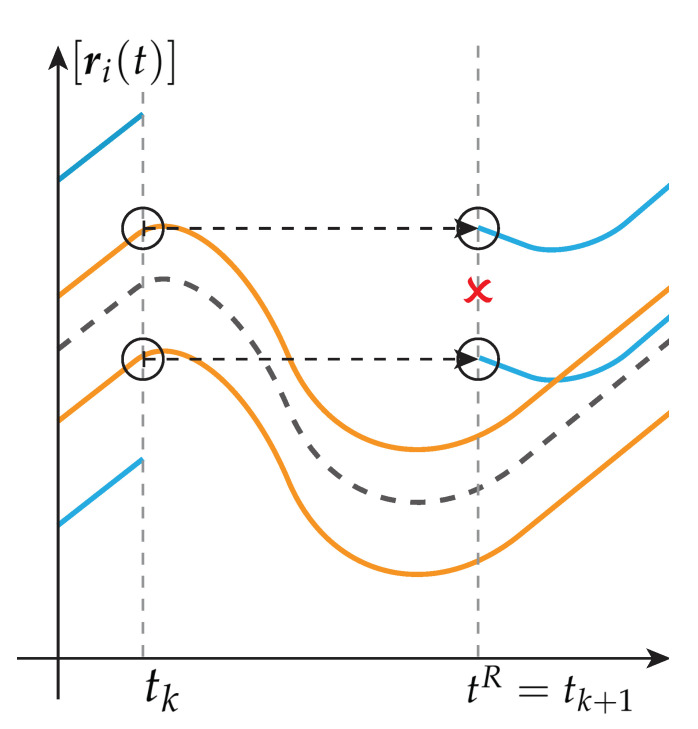
Re-initialization at time step $t^{R}=t_{k+1}$ with initial values obtained at $t_{k}$.

**Figure 3:**
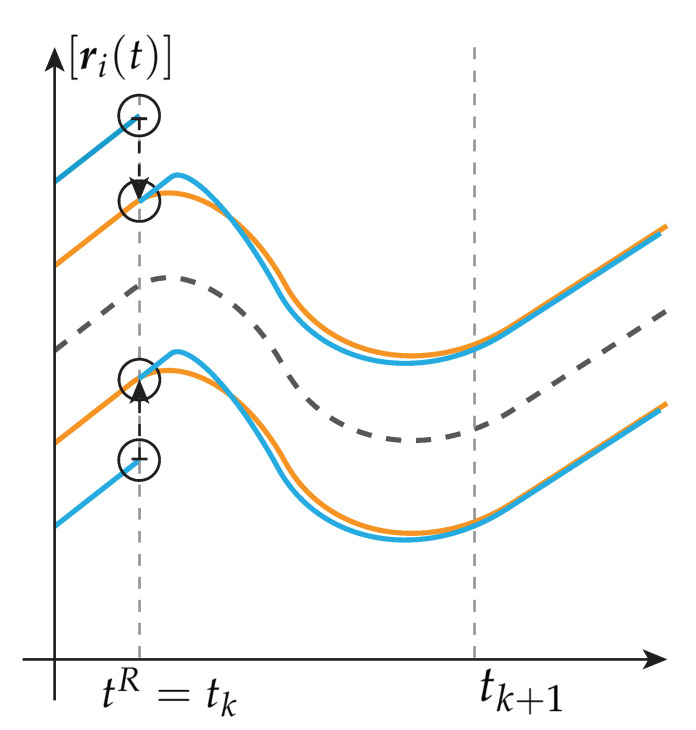
A whole re-initialization during time step $t^{R}=t_{k}$.

*To this, a principal behavior of two interval observers at re-initialization is shown in Figure 2 and Figure 3. For a finite small calculation step, it can be seen in Figure 2 that the two interval observers do not enclose the same set at $t_{k+1}$. In contrast, a valid interval estimation is guaranteed if the whole re-initialization algorithm is performed during the re-initialization time step $t^{R}$, which is presented in Figure 3.*

*Since Definitions 4 and 6 hold, every boundary of the interval observer bundle is used to calculate the best envelope after solving each initial value problem of the interval observers separately. Therefore, $l^{\left(𝓑\right)}\left(t\right)$, respectively the new initial values $\left[{\underline{r}}_{0}\left(t^{R}\right),\;{\bar{r}}_{0}\left(t^{R}\right)\right]$, depend on the calculated values of each interval observer. Hence, the query for re-initialization depends on every interval observer within the bundle. Consequently, the interval observers re-initialization depends on its own output values during the same time step leading to an algebraic loop [29].*

*Hence, initial values $x_{0}$ for re-initialization obtained at $t^{R}=t_{k}$ are needed to maintain guaranteed interval enclosures of the real values at subsequent time steps $t_{k+1}$.*

**Proof** **of** **Theorem** **1.** It is proven that any linear time-varying system has to be initialized within the same time step where the new initial values are obtained. Considered is a linear time-varying system:

(39) $$\dot{x}\left(t\right)=A\left(t\right)\;\cdot \;x\left(t\right)+B\left(t\right)\;\cdot \;u\left(t\right),$$

(40) $$y\left(t\right)=C\left(t\right)\;\cdot \;x\left(t\right),$$

with $A\left(t\right)\in R^{n\times n}$, $B\left(t\right)\in R^{n\times q}$, and $C\left(t\right)\in R^{p\times n}$, which are continuous real matrices. For each initial value $x\left(t_{0}\right)=x_{0}$, $u\left(t_{0}\right)=u_{0}$ and each continuous input function $u\left(t\right)\in R^{q}$, the system has a unique and continuously differentiable global solution Φ(t,x(t),u(t)) fulfilling the initial conditions $\Phi \left(0,x_{0},u_{0}\right)=x_{0}$. Furthermore, the output function $y\left(t\right)$ is continuous. Therefore, re-initialization at time step $t_{k+1}^{R}$, with a shifted initial value $x\left(t_{k}\right)=x_{0}\left(t_{k+1}\right)$ calculated during time step $t_{k}$ as proposed in [23] leads to the necessary initial value problem’s solution:

(41) $$\Phi \left(t_{k+1}^{R},x\left(t_{k}\right),u\left(t_{k}\right)\right)=x_{0}\left(t_{k+1}\right)$$

which does not satisfy the unique global solution Φ(t,x(t),u(t)) of the state space system (39) to (40) generally, since Equation (41) is not met for all $t\;\geq t_{0}$. □

**Remark** **6.** *In case of systems with relatively large time constants T, e.g., bioprocesses discussed in Moisan et al. [23], the requirement to execute the whole re-initialization process during one time step can be simplified under the assumption,*

(42) $$\Phi \left(t_{k+1}^{R},x_{0}\left(t_{k}\right),u_{0}\left(t_{k}\right)\right)\approx \Phi \left(t_{k+1},x_{0}\left(t_{k+1}\right),u_{0}\left(t_{k+1}\right)\right)$$

*with a sampling time Δt of the interval estimation algorithms that holds Δt≪T.*

**Remark** **7.** *In case of unstable systems with small time constants, a periodical re-initialization leads to areas, e.g., in between $t_{k}$ and $t_{k+1}$, wherein the behavior of the system states is not well defined leading to increasing calculation time or a complete termination of the program due to numerical overflow because of instable dynamics. Hence, in Definition 6, no condition subject to the re-initialization time $t^{R}$ contrary to Bernard and Gouzé [21] is made. Therefore, the procedure of re-initialization for systems with small time constants is discussed in detail.*

Thus, an algorithm for systems with relatively small time constants, which do not fulfill approximation (42), satisfying Theorem 1 has to be developed. To avoid computing intensive numerical algebraic loops or an analytical solution at each re-initialization time step $t^{R}$, resulting from the necessary calculations of Definition 4 and Proposition 1, the re-initialization is divided into sub tasks to solve the interval observer’s initial value problem, generating the boundaries $\underline{𝓑}\left(t\right)$ and $\bar{𝓑}\left(t\right)$, check the re-initialization conditions, and restart the concerned interval observers with new initial values given by $l^{\left(𝓑\right)}\left(t^{R}\right)$. The preservation of real-time capability is the question that arises.

To take advantage of the periodical re-initialization and to avoid numerical problems given by unstable framers, a re-initialization signal $\delta_{R}^{i}\left(t\right)$ activating the initialization of the integrators if $\delta_{R}^{i}\left(t\right)=1$ within the algorithms, is composed as follows:

(43) $$\delta_{R}^{i}\left(t\right)=\left\{\begin{array}{cc} 1 & \;\mathrm{for}\;t=k\;\cdot \;t_{r}\;\mathrm{with}\;k=1,\ldots ,\infty \in N,t_{r}\in R^{+}\\ 1 & \;\mathrm{if}\;\mathrm{any}\;|r_{i}{\left(t\right)|}_{j}\geq {\hat{r}}_{max}\;\mathrm{with}\;i=1,\ldots ,2n\\ 0 & \;\mathrm{otherwise} \end{array}\right.,$$

whereby, j=1,…,b, with the number of used interval observers *b* and $r_{i}\left(t\right)\in \left\{\bar{r}\left(t\right),\underline{r}\left(t\right)\right\}$. The threshold ${\hat{r}}_{max}$ is an approximation of expected estimated values limited by physical properties. The first condition in (43) is applied globally, but the second has to be checked with the states of each interval observer separately. If $\delta_{R}^{i}\left(t\right)=1$ for $\sum_{i}$, this interval observer gets re-initialized, the integrators are reset with the new initial values given by (38).

## 3. Modeling of an Induction Machine

To describe the induction machine’s behavior in the stationary reference frame, a parameter-varying state-space model based on the inverse gamma representation of the machine is used as applied in Li et al. [30] in a similar notation. The dynamics (IM) of the induction machine are given by:

(44) $$\dot{x}\left(t\right)=A\left(t\right)\;\cdot \;x\left(t\right)+B\;\cdot \;u\left(t\right),$$

(45) $$y\left(t\right)=C\;\cdot \;x\left(t\right),$$

with

(46) $$x\left(t\right)={\left[\begin{array}{cccc} i_{s,\alpha }\left(t\right) & i_{s,\beta }\left(t\right) & i_{\mu ,\alpha }\left(t\right) & i_{\mu ,\beta }\left(t\right) \end{array}\right]}^{⊺},$$

(47) $$u\left(t\right)=\left[\begin{array}{c} u_{s,\alpha }\left(t\right)\\ u_{s,\beta }\left(t\right) \end{array}\right],$$

(48) $$\begin{array}{c} A\left(t\right)=\left[\begin{array}{cccc} -\frac{R_{r}+R_{s}}{L_{\sigma s}} & 0 & \frac{R_{r}}{L_{\sigma s}} & \frac{\omega_{L}\left(t\right)\;\cdot \;L_{h}}{L_{\sigma s}}\\ 0 & -\frac{R_{r}+R_{s}}{L_{\sigma s}} & -\frac{\omega_{L}\left(t\right)\;\cdot \;L_{h}}{L_{\sigma s}} & \frac{R_{r}}{L_{\sigma s}}\\ \frac{R_{r}}{L_{h}} & 0 & -\frac{R_{r}}{L_{h}} & -\omega_{L}\left(t\right)\\ 0 & \frac{R_{r}}{L_{h}} & \omega_{L}\left(t\right) & -\frac{R_{r}}{L_{h}} \end{array}\right], \end{array}$$

(49) $$B={\left[\begin{array}{cccc} \frac{1}{L_{\sigma s}} & 0 & 0 & 0\\ 0 & \frac{1}{L_{\sigma s}} & 0 & 0 \end{array}\right]}^{⊺},$$

(50) $$C=\left[\begin{array}{cccc} 1 & 0 & 0 & 0\\ 0 & 1 & 0 & 0 \end{array}\right]$$

and

(51) $$\omega_{L}\left(t\right)=z_{p}\;\cdot \;\omega_{mech}\left(t\right).$$

The states $x\left(t\right)$ contain the measurable stator current components $i_{s,\alpha }\left(t\right)$ and $i_{s,\beta }\left(t\right)$ and the unmeasurable components of the magnetizing current $i_{\mu ,\alpha }\left(t\right)$ and $i_{\mu ,\beta }\left(t\right)$ while the input $u\left(t\right)$ contains the stator voltage components $u_{s,\alpha }\left(t\right)$ and $u_{s,\beta }\left(t\right)$. Five time-invariant parameters are included, namely the number of pole pairs $z_{p}$, the main inductance $L_{h}$, the stator leakage inductance $L_{\sigma s}$, the stator resistance $R_{s}$, and the rotor resistance $R_{r}$. The time-varying parameter $\omega_{mech}\left(t\right)$ is the mechanical angular velocity gained by the differentiation of measured mechanical angle.

The torque of the induction machine is given in Quang et al. [31] by:

(52) $$M\left(t\right)=\frac{3}{2}\;\cdot \;z_{p}\;\cdot \;𝓘m\left\{\Psi_{s}^{*}\left(t\right)\;\cdot \;i_{s}\left(t\right)\right\}.$$

Applying the equation:

(53) $$\begin{array}{c} \Psi_{s}^{*}\left(t\right)=L_{h}\;\cdot \;i_{r}^{*}\left(t\right)=L_{h}\;\cdot \;\left(i_{\mu }\left(t\right)-i_{s}^{*}\left(t\right)\right) \end{array}$$

as well as:

(54) $$\begin{array}{cc} i_{s}\left(t\right) & =i_{s,\alpha }\left(t\right)+j\;\cdot \;i_{s,\beta }\left(t\right) \end{array}$$

and

(55) $$\begin{array}{cc} i_{\mu }\left(t\right) & =i_{\mu ,\alpha }\left(t\right)+j\;\cdot \;i_{\mu ,\beta }\left(t\right) \end{array}$$

yields the torque of the induction machine:

(56) $$M\left(t\right)=\frac{3}{2}\;\cdot \;z_{p}\;\cdot \;L_{h}𝓘m\left\{i_{s,\beta }\;\cdot \;i_{\mu ,\alpha }-i_{s,\alpha }\;\cdot \;i_{\mu ,\beta }\right\}.$$

Hereby, complex conjugate numbers are marked with ${\left(.\right)}^{*}$ and the imaginary unit j is considered as well as the operator Im{(.)} selecting the value of the imaginary part of the complex number. Furthermore, the interval representation of the torque is calculated using basic interval arithmetic operations as defined in Jaulin et al. [13].

## 4. The Design and Implementation of the Bundle of Interval Observers for the Induction Machine

In this section, the preliminaries presented in the previous sections are used and the methods are applied for the design and implementation of the bundle of observers for the induction machine. The composition of the reduced-order hybrid interval observers into a bundle of observers and the re-initialization process as well as the parametrization of the observer’s gain are presented. The number of used reduced-order hybrid interval observer is defined by the suggested optimization strategy. A general design procedure closing this section summarizes the algorithms presented in this paper.

### 4.1. The Re-Initialization Process

For the re-initialization signal $\delta_{R}^{i}\left(t\right)$, the two parameters $t_{r}$ and ${\hat{r}}_{max}$ have to be chosen. Firstly, the periodical re-initialization time is chosen to $t_{r}=0.25s$. Since there is no information given about the behavior of the transient phase of the magnetizing current, this value is a first guess and has to be adjusted with simulations or experiments.

The maximum expected value is derived from a physical point of view. Given the rated current of the induction machine $i_{rc}=21A$, an upper limit of expected currents ${\hat{r}}_{max}$ can be approximated using the relationship of the inrush current, which is about seven to eight times higher than the rated current [32]. By representation in αβ-coordinates, the in-rush current has to be transformed using the Clarke transformation (3). Summarized, in case of the induction machine presented in this paper, the upper boundary of expected values is established as:

(57) $${\hat{r}}_{max}=224A.$$

**Figure 4:**
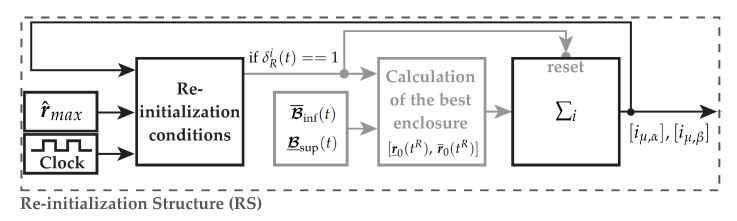
Structure and signal flow of the re-initialization process of one reduced-order hybrid interval observer. The gray part of the algorithm is executed only if the condition $\delta_{R}^{i}\left(t\right)==1$ is fulfilled.

Finally, the structure of the re-initialization algorithm under consideration of Lemma 1 is obtained as depicted in Figure 4. In this figure, the black path is processed continuously during runtime. Only if one of the re-initialization conditions is true, the gray part of the algorithm gets executed resulting in an initialization of the integrator. Within this figure, the remaining parts of the reduced-order interval observer, see Figure 1, are not shown.

### 4.2. The Interval Observer Gain

For the design of the reduced-order hybrid interval observer, the observer gain L(t) using (15) and satisfying positivity as well as stability of the estimation errors is further named $L_{f}\left(t\right)$ and given by:

(58) $$\begin{array}{c} L_{f}\left(t\right)=\left[\begin{array}{cc} l_{f,11}\left(t\right) & l_{f,12}\left(t\right)\\ l_{f,21}\left(t\right) & l_{f,22}\left(t\right) \end{array}\right]=\left(A_{22}\left(t\right)-\left[\begin{array}{cc} f_{11} & f_{12}\\ f_{21} & f_{22} \end{array}\right]\right)A_{12}^{-1}\left(t\right). \end{array}$$

Hereby, $f_{ij}\in \theta_{f}=\left[f_{11},f_{12},f_{21},f_{22}\right]$ with i,j∈{1,2} are the elements of the resulting system matrix F, see (15), utilized as design parameters $\theta_{f}$ of the interval observers. Furthermore, because of the time-variant matrices $A_{22}\left(t\right)$ and $A_{12}^{-1}\left(t\right)$, the observer gain $L_{f}\left(t\right)$ is dependent on $\omega_{L}\left(t\right)$. The precondition that the matrix $A_{12}\left(t\right)$ is invertible for all $t\;\geq t_{0}$ is always met for the induction machine. When applying to other technical systems, this condition has to be checked to obtain a general formula for the observer gain $L_{f}\left(t\right)$ yielding a constant system matrix (15).

Secondly, an observer gain independent of the system matrix may be desired. In the following, such an observer gain is denoted as:

(59) $$L_{l}\left(t\right)=\left[\begin{array}{cc} l_{l,11}\left(t\right) & l_{l,12}\left(t\right)\\ l_{l,21}\left(t\right) & l_{l,22}\left(t\right) \end{array}\right],$$

with the elements $l_{l,ij}\in \theta_{l}=\left[l_{11},l_{12},l_{21},l_{22}\right]$ with i,j∈{1,2}. Given the observer gain $L_{l}\left(t\right)$, stability of the error dynamics is not ensured because the resulting system matrix F(t) is time-varying and hence, the matrix $\tilde{F}$ of (31) is not Hurwitz in every case. However, with this observer gain a framer can be set up.

### 4.3. Parametrization of the Observers

An optimization algorithm is proposed that determines configurations for the interval observers in order to minimize the interval widths for a feasible operation area of the time-varying parameter $\omega_{L}\left(t\right)$. Additionally, unstable configurations are a result of this procedure which improve the interval widths further. For the implementation of the optimization’s results, lookup tables (LUT) followed by the adaptive observer gain are designed.

Given the basic idea of a bundle of interval observers, it is useful to optimize the estimation of each interval in $\left[r\left(t\right)\right]$ separately. In case of the induction machine these are the intervals for the magnetizing currents:

(60) $$\begin{array}{c} \left[{\bar{r}}^{⊺}\left(t\right),\;{\underline{r}}^{⊺}\left(t\right)\right]=\left[{\bar{i}}_{\mu ,\alpha }\left(t\right),\;{\bar{i}}_{\mu ,\beta }\left(t\right),\;{\underline{i}}_{\mu ,\alpha }\left(t\right),\;{\underline{i}}_{\mu ,\beta }\left(t\right)\right]. \end{array}$$

Subsequently, there width is called $l_{\alpha }\left(t\right)$ and $l_{\beta }\left(t\right)$ and calculated using:

(61) $$\begin{array}{c} {\left[l_{\alpha }\left(t\right),\;l_{\beta }\left(t\right)\right]}^{⊺}={\left[{\bar{i}}_{\mu ,\alpha }\left(t\right)-{\underline{i}}_{\mu ,\alpha }\left(t\right),\;{\bar{i}}_{\mu ,\beta }\left(t\right)-{\underline{i}}_{\mu ,\beta }\left(t\right)\right]}^{⊺}. \end{array}$$

Hence, half of the interval observers within the bundle are optimized to shrink the interval width $l_{\alpha }\left(t\right)$ and the other half the interval width $l_{\beta }\left(t\right)$.

Furthermore, both introduced observer gains (58) and (59) should be utilized to achieve optimal results. On the one hand, this is reasonable since the observer gain (59) is neither influenced by $\omega_{L}\left(t\right)$, nor G(t) by ${\dot{\omega }}_{L}\left(t\right)$ (see Equation (24)). In that case: the observer gain is time-varying and the estimation interval is enlarged because of additional interval calculations resulting in an amplification of the wrapping effect [33]. On the other hand, the information of the angular velocity is useful to minimize the interval widths with (58), because the operation point is known and an optimal gain can be calculated offline.

Unfortunately, with a time-varying $\omega_{L}\left(t\right)$ for both sets of possible observer parameters $\theta_{l}\left(t\right)=\left[l_{11}\left(t\right),l_{12}\left(t\right),l_{21}\left(t\right),l_{22}\left(t\right)\right]$ and $\theta_{f}\left(t\right)=\left[f_{11}\left(t\right),f_{12}\left(t\right),f_{21}\left(t\right),f_{22}\left(t\right)\right]$, whereby, both sets can be constant or time varying, it is not possible to find the minimal enclosures of the interval error estimation (30) analytically. The reasons are complex dependencies of the matrices of (30) to obtain zeros of its first derivation analytically in order to minimize the influence of the observer gain, see Appendix A for a full representation of the system matrices. Hence, the interval widths are minimized numerically based on scenarios of $\omega_{L}\left(t\right)$ considering a variety of observer gains $\theta_{l}\left(t\right)$ and $\theta_{f}\left(t\right)$ within the bundle of interval observers.

**Figure 5:**
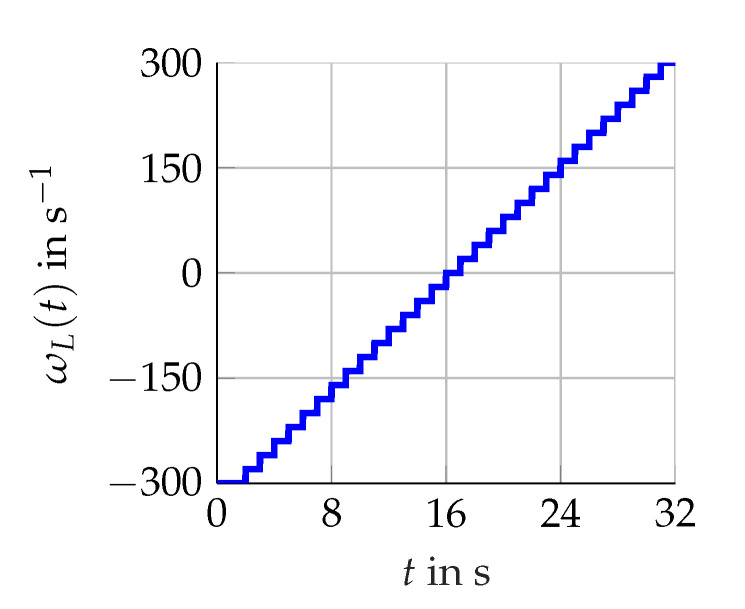
Scenario A for $\omega_{L}\left(t\right)$ representing constant operating points (revolutions per second).

**Figure 6:**
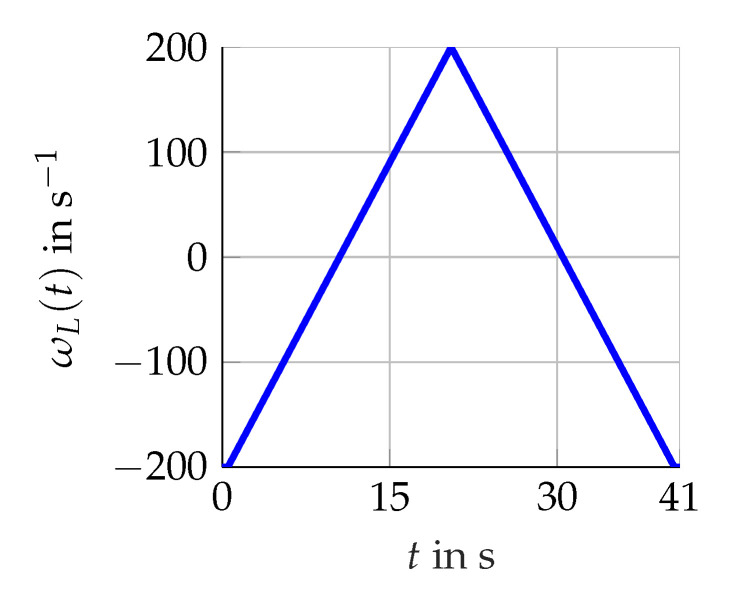
Scenario B for a slowly changing $\omega_{L}\left(t\right)$ (revolutions per second).

**Figure 7:**
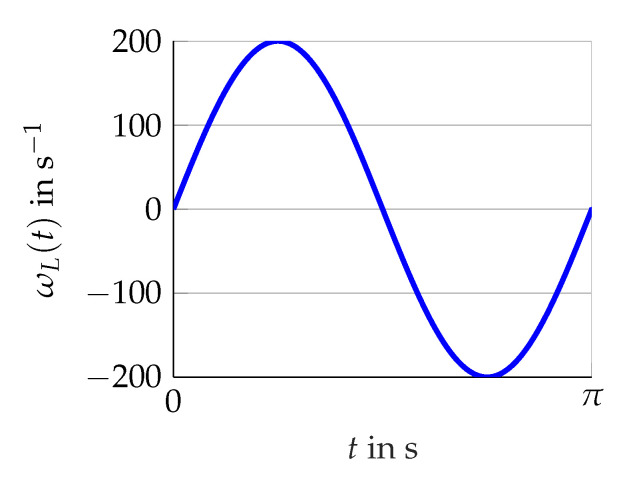
Scenario C for more rapid changes of $\omega_{L}\left(t\right)$ (revolutions per second).

The three scenarios shown in Figure 5, Figure 6 and Figure 7 are considered. Firstly, various constants (scenario A) of $\omega_{L}\left(t\right)$ are utilized. Secondly, the rising and falling edges of a linear (scenario B) as well as a sinus function (scenario C) representing $\omega_{L}\left(t\right)$ with either ${\dot{\omega }}_{L}\left(t\right)\geq 0$ or ${\dot{\omega }}_{L}\left(t\right)<0$. For these scenarios, the torque remains constant $M_{set}\left(t\right)=4Nm$.

Given these scenarios, the number of interval observers within the bundle are defined. For each magnetizing current $l_{\alpha }\left(t\right)$ and $l_{\beta }\left(t\right)$ an interval observer is needed for the scenario (Figure 5) and two interval observers for both directions of the derivatives ${\dot{\omega }}_{L}\left(t\right)$ for Figure 6 and Figure 7. Given two different approaches for observer gain (58) and (59), the total number of interval observers within the bundle is 20. This number can be increased with more scenarios or additional observer gains. Within this contribution, the simulation of the whole procedure is done with these 20 observers representing each proposed combination of parameters, scenarios, and signs of the time-varying parameters. For each combination of scenario, sign of ${\dot{\omega }}_{L}\left(t\right)$ and set of observer gains $\theta_{l}\left(t\right)$ or $\theta_{f}\left(t\right)$ one optimization is processed leading to one parametrization for the specific combination. Obviously, more scenarios or distinct ranges of $\omega_{L}\left(t\right)$ can be determined in order to polish the interval observation, but, this should be done with the requirements of an actual task specifying the desired operation range of the induction machine.

For the implementation of the adaptive observer structure, the scenarios for $\omega_{L}\left(t\right)$ are divided into parts representing different operation areas. For each part, the optimization is solved offline with the given scenarios. The resulting parameters are stored in lookup tables (LUT) which can then be called during online operation. The necessary values u(t) and y(t) for the optimization are calculated with the scenario and the induction machine’s model, see Section 3, in advance. These data are divided into *m* sections resulting in $\omega_{L}\left(\Delta t_{i}\right)$, $u(\Delta t_{i})$, and $y(\Delta t_{i})$. In each section, $\Delta t_{i}=t_{i+1}-t_{i}$ with i=0,1,…,m−1∈N, a feasible set of parameters is searched. The edges of these sections are the supporting points of the lookup tables.

Given this setup, either the parameters $\theta_{l}$ or $\theta_{f}$ are used in order to minimize $l_{\alpha }\left(t\right)$ or $l_{\beta }\left(t\right)$. For each section $\Delta t_{i}$ in the scenarios, one optimization is performed in order to obtain the optimal values $\theta_{l}^{*}$ and $\theta_{f}^{*}$ for this section. The objective functions (62) are minimized in order to obtain optimal parameters for the associated operating area. Hereby *i* specifies the requested set of parameters by the lookup table which is passed to the observer gain during online operation.

(62) $$\begin{array}{c} \begin{array}{cc} J_{\alpha }\left(\theta_{l}^{*}\right) & =\min\int_{t_{i}}^{t_{i+1}}l_{\alpha }\left(\theta_{l}^{i},\left[u\left(\tau \right)\right],\left[y\left(\tau \right)\right],\omega_{L}\left(\tau \right),\tau \right)d\tau ,\\ J_{\alpha }\left(\theta_{f}^{*}\right) & =\min\int_{t_{i}}^{t_{i+1}}l_{\alpha }\left(\theta_{f}^{i},\left[u\left(\tau \right)\right],\left[y\left(\tau \right)\right],\omega_{L}\left(\tau \right),\tau \right)d\tau ,\\ J_{\beta }\left(\theta_{l}^{*}\right) & =\min\int_{t_{i}}^{t_{i+1}}l_{\beta }\left(\theta_{l}^{i},\left[u\left(\tau \right)\right],\left[y\left(\tau \right)\right],\omega_{L}\left(\tau \right),\tau \right)d\tau ,\\ J_{\beta }\left(\theta_{f}^{*}\right) & =\min\int_{t_{i}}^{t_{i+1}}l_{\beta }\left(\theta_{f}^{i},\left[u\left(\tau \right)\right],\left[y\left(\tau \right)\right],\omega_{L}\left(\tau \right),\tau \right)d\tau . \end{array} \end{array}$$

It should be noted that there are no constraints given for the parameters. This is intended to ensure that unstable configurations can be set up. With the presented re-initialization, such unstable framers tighten the interval estimation further. To avoid problems with high numerical values due to unstable eigenvalues, which may lead to the termination of the optimization, one stable interval observer runs in parallel, as it would be within a practical application, but, without impact on the optimization process. Furthermore, this is necessary so as to monitor and re-initialize the unstable framer (procedure as presented in Section 4.1).

Finally, a basic sectioning algorithm solving the optimization numerically is chosen [34]. With the assumption of multiple local minima, the algorithm should be able to bypass problems like jumping between valleys or a low convergence rate. It is expected that the algorithm remains within such a local minimum, which is inthe case of this paper, a suitable approach that is sufficient to show the effectiveness of the general approach.

### 4.4. Adaptive Observer Gain Using Lookup Tables

Before the bundle of observers is set up, some preliminaries and requirements have to be reconsidered. Given Equation (24) and the proof of Proposition 1, the elements of the observer gains as well as their derivatives in (64) have to be continuous. These derivatives are given by:

(63) $$\begin{array}{cc} \frac{d}{dt}L\left(\theta_{f}\left(t\right),t\right) & =\dot{L}\left({\dot{\theta }}_{f}\left(t\right),\theta_{f}\left(t\right),t\right) \end{array}$$

and

(64) $$\begin{array}{cc} \frac{d}{dt}L\left(\theta_{l}\left(t\right),t\right) & =\dot{L}\left({\dot{\theta }}_{l}\left(t\right),\theta_{l}\left(t\right),t\right). \end{array}$$

Equation (63) is presented in detail in Appendix A, the derivative of (64) can only be approximated numerically since there is no functional connection of the change between two consecutive values of $\theta_{l}\left(t\right)$. Hence, if an observer, using the optimal configurations stored in the lookup tables, is to be implemented, the scheduling of L(t) and $\dot{L}\left(t\right)$ is to be designed such that continuity is assured.

### 4.5. Parameter Switching Functions

With the results of Section 4.3, a switching function has to take different operating areas into account. Hereby *i* specifies the requested parameter by the lookup table (LUT). If the measured value of the angular velocity lies within a specific section $\omega_{L}\left(\Delta t_{i}\right)$, the matching observer configurations $\theta_{l}^{i}\left(\Delta t_{i}\right)$ or $\theta_{f}^{i}\left(\Delta t_{i}\right)$ are retrieved from the lookup table and passed to the observer. These changes, comparable to step functions, are not continuous, thus, they give no satisfaction on the necessary continuous derivatives (63) and (64). Furthermore, because of the necessary continuous derivatives ${\dot{\theta }}_{l}\left(t\right)$ and ${\dot{\theta }}_{f}\left(t\right)$, interpolation with spline methods or polynomials is difficult to set up and computationally intensive, especially for online operation. The condition for a continuously differentiable change of parameters is met if:

(65) $$\begin{array}{c} {\ddot{\theta }}_{l,f}^{i}\left(t_{k}\right)={\ddot{\theta }}_{l,f}^{i+1}\left(t_{k+1}\right) \end{array}$$

is satisfied. To meet the requirements, the second order linear time invariant (LTI) transfer function (66) is introduced, in order to filter the change of the parameters.

(66) $$\begin{array}{cc} G_{\eta }\left(s\right) & =\frac{1}{{\left(s+\eta \right)}^{2}}=\frac{MM1\;/\;\eta^{2}}{MM1\;/\;\eta^{2}s^{2}+MM2\;/\;\eta s+1} \end{array}$$

With two identical time constants η, which can be used as tuning parameters, an overshoot is prevented. This is reasonable with the proposed optimization that generates configurations which are approximately local minimizers in between two supporting points of $\omega_{L}\left(t\right)$. With the transfer function (66) and an additional auxiliary state $z_{1}\left(t\right)$ to monitor condition (65) the state-space system (67) achieving a continuous differentiable adaptive gain is set up.

(67) $$\begin{array}{c} \begin{array}{cc} \left[\begin{array}{c} {\dot{z}}_{1}\left(t\right)\\ {\dot{z}}_{2}\left(t\right)\\ {\dot{z}}_{3}\left(t\right) \end{array}\right]\; & =\left[\begin{array}{ccc} 0 & 1 & 0\\ 0 & 0 & 1\\ 0 & -1\;/\;\eta^{2} & -2\;/\;\eta \end{array}\right]\left[\begin{array}{c} z_{1}\left(t\right)\\ z_{2}\left(t\right)\\ z_{3}\left(t\right) \end{array}\right]+\left[\begin{array}{c} 0\\ 0\\ 1 \end{array}\right]\theta^{i+1}\\ \left[\begin{array}{c} \theta_{i}\left(t\right)\\ {\dot{\theta }}_{i}\left(t\right)\\ {\ddot{\theta }}_{i}\left(t\right) \end{array}\right]\; & =\left[\begin{array}{ccc} 0 & -1\;/\;\eta^{2} & 0\\ 0 & 0 & -2\;/\;\eta \\ 1 & 0 & 0 \end{array}\right]\left[\begin{array}{c} z_{1}\left(t\right)\\ z_{2}\left(t\right)\\ z_{3}\left(t\right) \end{array}\right]\\ \;\mathrm{with}\;z_{0} & =\theta^{i}\;\cdot \;\eta^{2} \end{array} \end{array}$$

**Figure 8:**
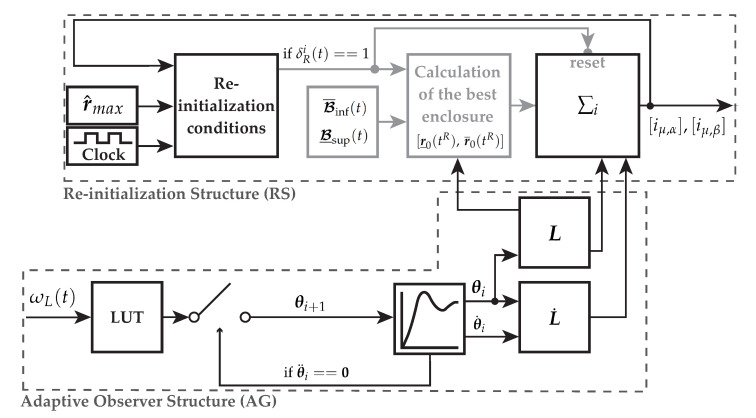
Extensions of the interval observer: Re-initialization structure (RS) and the adaptive observer gain (AG) selecting the optimal observer parametrization θ from a lookup table (LUT) depending on $\omega_{L}\left(t\right)$. After the LTI (linear time invariant) switching function (67), the observer gain L as well as its derivative $\dot{L}$ are set up for the reduced-order interval observer.

The state $z_{1}\left(t\right)$ is necessary since the lookup table may request new observer gains during the transient phase for the previous parameter set leading to noncontinuous behaviors. Hence, a change of gains is possible as long as ${\ddot{\theta }}_{i}\left(t\right)={\ddot{\theta }}_{i+1}\left(t\right)=0$ with $z_{1}\left(t\right)={\ddot{\theta }}_{i}\left(t\right)$. Otherwise the locus of ${\dot{z}}_{3}\left(t\right)$ and ${\dot{z}}_{2}\left(t\right)$ is not continuous. In Figure 8, the structure of the adaptive observer gain (AG) as well as the re-initialization (RS) is shown.

### 4.6. Set Up the Error Model

It is not sufficient to set up an arbitrary error model when using interval observers at a plant where measurement data are obtained using sensors and control variables are uncertain. Therefore, a feasible error model for the input voltages $u_{u,v,w}\left(t\right)$ as well as the stator currents $i_{s,\alpha }\left(t\right)$ and $i_{s,\beta }\left(t\right)$ have to be selected.

The measurement error of the stator currents is provided by the sensor manufacturer. Unfortunately, the error model for the input voltages is not given directly. Though, using the interval model of an inverter and the knowledge about the uncertain DC link voltage leads to the representation of the input voltages as intervals. A detailed interval model of the chosen inverter model is presented in Krebs et al. [35] which completes the error model for the interval observers. The error model of the setup is given as follows:

(68) $$\begin{array}{c} {𝓘}_{\xi }=\left\{{\left[U_{DC}\right]}^{0.48V},{\left[y\left(t\right)\right]}^{0.5A}\right\}. \end{array}$$

Herein, $\left[U_{DC}\right]$ is the DC link voltage’s uncertainty. The parameters are assumed to be exactly known.

(69) $$\begin{array}{c} {𝓘}_{\kappa }=\emptyset \end{array}$$

### 4.7. Composition of the Estimation Setup

Finally, the bundle of interval observers is given by (70).

(70) $$\begin{array}{c} 𝓑:\left\{\begin{array}{cc} \; & \left\{\sum_{1},\sum_{2},\ldots ,\sum_{20}\right\}\\ \; & {𝓘}_{\kappa }=\emptyset \\ \; & {𝓘}_{\xi }=\left\{{\left[U_{DC}\right]}^{0.48V},{\left[y\left(t\right)\right]}^{0.5A}\right\}\\ \; & \left(\mathrm{IM}\right) \end{array}\right.. \end{array}$$

**Figure 9:**
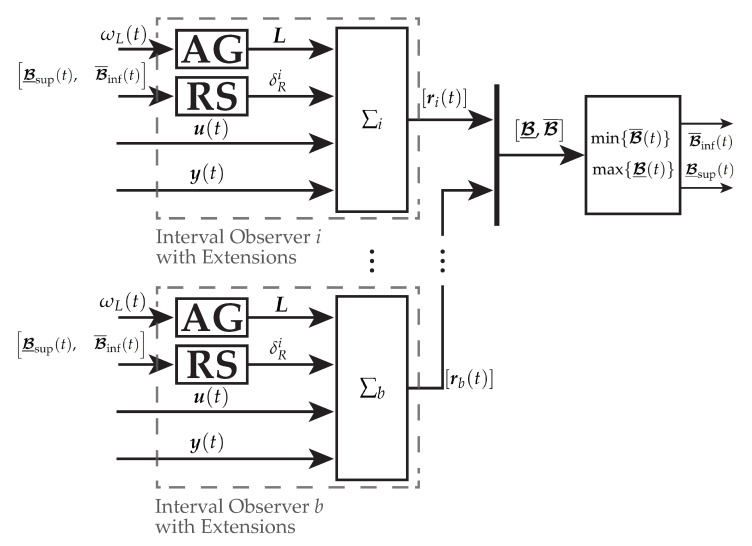
Structure of the interval observer bundle 𝓑. Hereby, $\sum_{i}$ are the reduced order interval observers considered within the bundle and $\left[r_{i}\left(t\right)\right]$ are the estimated interval values. The best envelopes $l^{\left(𝓑\right)}\left(t\right)$ are calculated with ${\bar{𝓑}}_{\inf}\left(t\right)$ and ${\underline{𝓑}}_{\sup}\left(t\right)$, see Definition 4. Adaptive observer parameters are obtained based on $\omega_{L}\left(t\right)$ with the adaptive observer gain (AG) and re-initialization conditions are monitored within the re-initialization structure (RS) providing the re-initialization signal $\delta_{R}^{i}$.

The hybrid interval observers $\sum_{i}$ with Equations (21)–(26) estimating the magnetizing current components $r\left(t\right)={\left[i_{\mu ,\alpha }\left(t\right),i_{\mu ,\beta }\left(t\right)\right]}^{⊺}$ as well as the extensions, presented in the previous sections, are shown in Figure 1. The narrowest envelopes are calculated using Definition 4. Each interval observer is supplemented with the re-initialization procedure (RS) as presented in Section 4.1 as well as a lookup table (LUT) together with the adaptive observer gain (AG) using the optimization results given by Section 4.3 as shown in Figure 8. The signal-flow graph is presented in Figure 9.

### 4.8. Summary of the Design Approach

**Figure 10:**
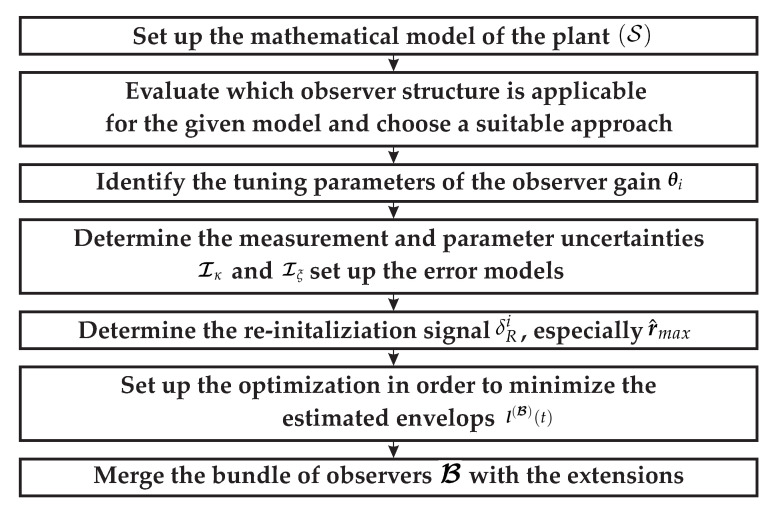
Flowchart of the design procedure.

In Figure 10, a flowchart for the design procedure is presented. Given the technical representation of the problem to be observed, a suitable mathematical model (S) must be established (cf. Section 3). With this model the interval observer architecture can be chosen, for example the reduced-order hybrid interval observer presented in this paper (see Section 2.3). Given the structure of the interval observer, the tuning parameters can be identified (cf. Section 4.2). These parameters are used for offline optimization to get a suitable parameterization for each operating area of interest (see Section 4.3 and Section 4.4). With a priori knowledge about the system’s time constants and an approximation for the maximum expected values ${\hat{r}}_{max}$, the re-initialization signal is to be determined. The error model is assembled with the information about the sensor and parameter uncertainties ${𝓘}_{\kappa }$ and ${𝓘}_{\xi }$ (cf. Section 4.6). Finally, the bundle of interval observer can be implemented as presented within the previous sections.

## 5. Results

The simulation and experimental results are obtained by considering an induction machine with a rated power of 2 kW, a rated torque of 6.3 Nm, and a rated speed of 3000 $\frac{1}{\min}$. The numerical values of the parameters listed in Table 1 are assumed to be known exactly. A lookup table for the torque control as well as a cascaded PI controller for the current control followed by an ideal voltage source inverter, with a DC link voltage of 48 V arrange the control system of the induction machine. As load, a speed regulated synchronous machine with a maximum torque of 32 Nm is used. The angular velocity $\omega_{mech}\left(t\right)$ is obtained by an incremental encoder and assumed to be known exactly. Within the simulation, the setpoint of the output torque is given as $M_{set}\left(t\right)$ and Equation (56) is used to calculate the output torque of the induction machine. Hence, only the interval inclusion of the measured torque $M_{mv}\left(t\right)$ against the calculated interval with Equation (56) and the estimations are presented. Within the experimental setup, the input voltages u(t) can not be measured directly. Therefore, these values have to be calculated with the controllers output signals and the ideal model of a voltage source converter leading to the necessary input voltages u(t).

**Figure 11:**
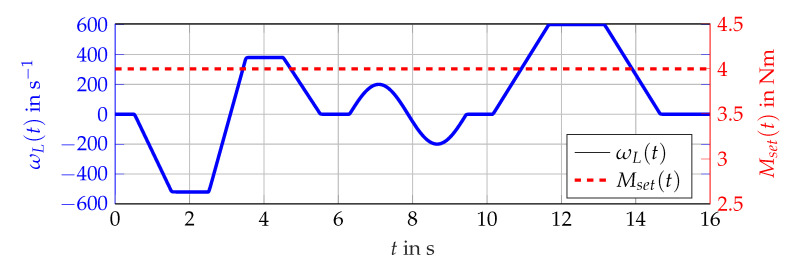
Used scenario for the simulation. $M_{set}\left(t\right)$ (red, dashed) and $\omega_{L}\left(t\right)$ (blue, solid).

**Figure 12:**
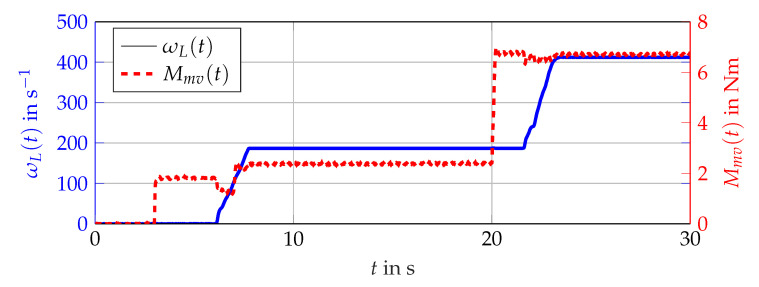
Scenario given by measurement data obtained at the test bench. Measured torque $M_{mv}\left(t\right)$ (red, dashed) and angular velocity $\omega_{L}\left(t\right)$ (blue, solid).

**Figure 13:**
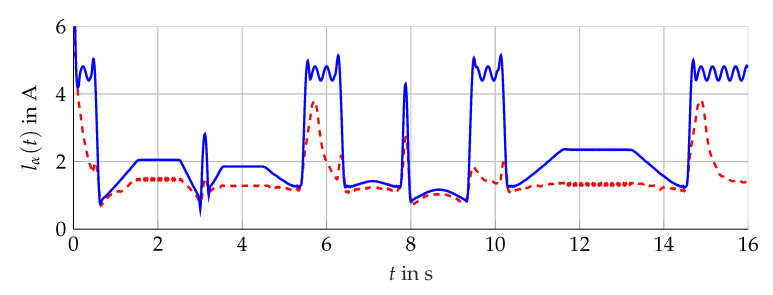
Interval width $l_{\alpha }\left(t\right)$ of the bundle of observers 𝓑 (red) compared with the single interval observer $\sum_{\mathrm{REF}}$ (blue).

**Figure 14:**
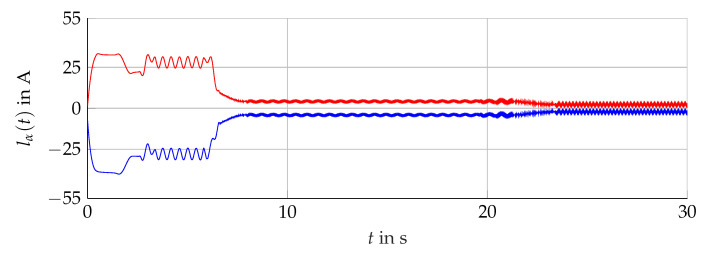
Interval boundaries ${\bar{i}}_{\mu ,\alpha }\left(t\right)$ (red) and ${\underline{i}}_{\mu ,\alpha }\left(t\right)$ (blue) of the bundle of observers 𝓑 of the scenario with measurement data.

**Figure 15:**
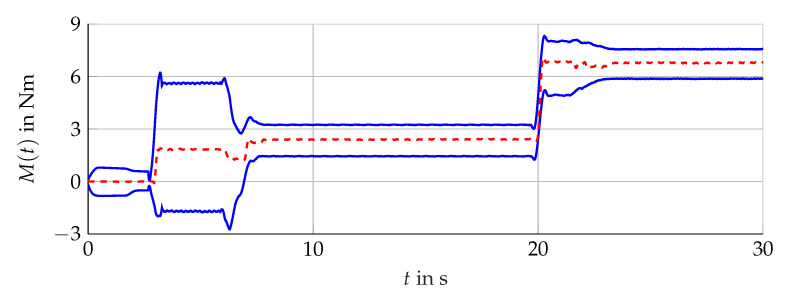
Torque interval boundaries $\bar{M}\left(t\right)$ and $\underline{M}\left(t\right)$ (blue, solid) calculated with ${\bar{i}}_{\mu ,\alpha }\left(t\right)$ and ${\underline{i}}_{\mu ,\alpha }\left(t\right)$ of 𝓑 (see Figure 14) compared with the measured torque $M_{mv}\left(t\right)$ (red, dashed).

The simulation scenario is shown in Figure 11. For the analysis with measurement data, the scenario presented in Figure 12 is considered. The comparison of the bundle of observers against the benchmark interval observer $\sum_{\mathrm{REF}}$ given by [10] is shown in Figure 13 (simulation) and Figure 14 (measurement data). With (56) and the measured output torque, the validation is presented in Figure 15.

The results are presented for the interval estimation of $i_{\mu ,\alpha }\left(t\right)$. Qualitatively, the same results are obtained for $i_{\mu ,\beta }\left(t\right)$.

### 5.1. Interval Width Improvement

**Figure 16:**
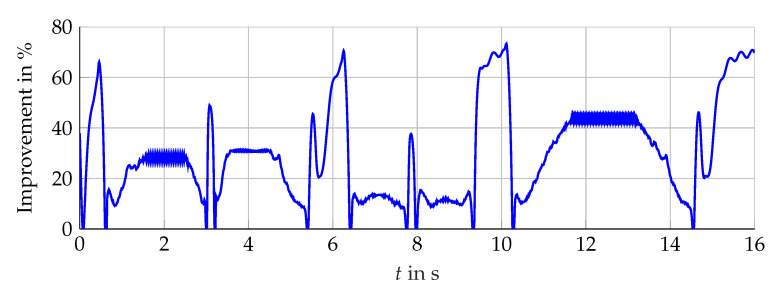
Relative interval width improvement in % with respect to $\sum_{\mathrm{REF}}$.

**Figure 17:**
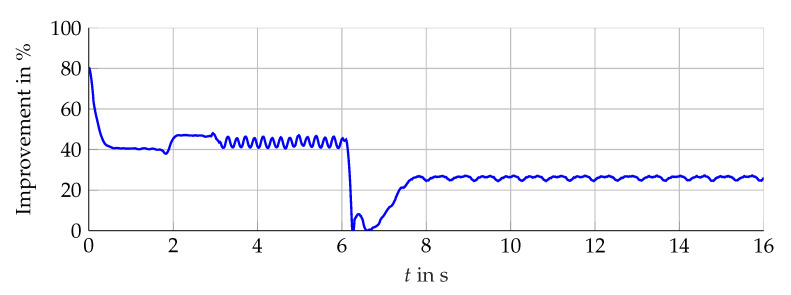
Relative interval width improvement in % with respect to $\sum_{\mathrm{REF}}$.

**Figure 18:**
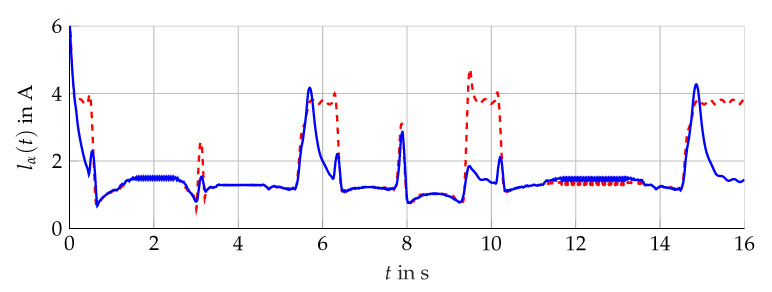
Interval width $l_{\alpha }\left(t\right)$ of the bundle of observers only with configurations $\theta_{f}\left(t\right)$ (red, dashed) compared with $\theta_{l}\left(t\right)$ (blue, solid).

Compared with the single interval observer, the bundle is able to suppress the initial overshoot and to achieve a fast convergence for different operation areas. The narrowing of the envelope $l_{\alpha }\left(t\right)$ shows significant improvement presented in Figure 16 (simulation) and Figure 17 (measurement data). Furthermore, the influence of the angular velocity’s amplitude during periods with constant $\omega_{L}\left(t\right)$ is nearly decoupled from the resulting interval width. The bundle of observers shows its full potential in the neighborhood around $\omega_{L}\left(t\right)=0$. In this operation area, the influence of the interval arithmetic operations is decisive but minimized with the optimized observer configurations. Nevertheless, the combination of rapid changes and zero crossings of $\omega_{L}\left(t\right)$ challenge the interval observers for the induction machine. Hereby, the interval observers implemented with (59) are able to reduce the interval width, which is presented in Figure 18, but still, a temporarily widening can not be avoided with the established configuration.

**Figure 19:**
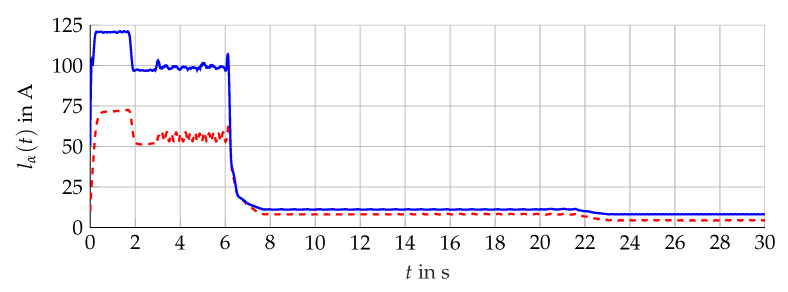
Interval width $l_{\alpha }\left(t\right)$ of the bundle of observers 𝓑 (red, dashed) compared with the single interval observer $\sum_{\mathrm{REF}}$ (blue, solid) of the scenario with measurement data.

A similar improvement is obtained with measurement data, see Figure 19. However, the resulting interval width is not suitable, which can mainly be attributed to a too large input uncertainty, due to the interval model of the inverter that is adding additional interval calculations within the chain of the estimation algorithm. Hereby, each zero crossing of the stator currents within the used interval model of the inverter contributes the deterioration of the interval width. The principle improvement of the guaranteed state estimation with measurement data is shown.

The validation of the reduced-order interval observer and the observer bundle is successful. The calculated interval of the output torque follows the measured torque’s dynamics and enclosures the real value for the whole simulation (see Figure 15).

### 5.2. Reinitalization & Unstable Configurations

Firstly, the periodical re-initialization has not shown improvements on the interval widths. Hence, the re-initialization process is mainly driven by the second case of (43). Nevertheless, the re-initialization process is used to take advantage of unstable configurations and is necessary for the implementation of the unstable interval observers.

The presented optimization achieves two different types of results for the parameters $\theta_{l}$ and $\theta_{f}$:

(i) The eigenvalues of the resulting system matrices are negative, which also yields stable and bounded error dynamics in case of $\theta_{f}$ together with Proposition Section 2.3;
(ii) Considering the optimization and the results with parameters $\theta_{f}$ it can be observed that the optimization algorithm shifts one off-diagonal element $f_{12}$ or $f_{21}$ of the system matrix to zero. The matching main diagonal element $f_{11}$ or $f_{22}$ then results in an unstable eigenvalue. Hence, the dynamics of this unstable eigenvalue does not affect the dynamics of the stable eigenvalue. The remaining optimization parameters, in case of the induction machine three, are used to minimize the interval width. Since the optimization and estimation is done separately for each unmeasurable state, this procedure leads to appropriate results. Further improvements could be made by setting one off-diagonal element a priori to zero and starting the optimization using this knowledge about the behavior and the considered scenario.

### 5.3. Real-Time Capability

The interval observer bundle presented in this paper was successfully tested at the test bench sketched in Section 5 and [10]. For the investigation of the discussed methods, the number of implemented interval observers within the bundle was gradually increased until the real-time controller (dSPACE ds1005 Controller Board) was unable to perform the algorithm in real-time. It resulted in a total number of 15 reduced-order hybrid interval observers with the whole extensions established in this paper capable of running in parallel. Hereby, none of the possible combinations with the prior proposed 20 interval observers are tested. Such a procedure needs to be done if the bundle of observers is applied to a specific technical system, whereby, the overall performances needs to be optimal.

For the experimental setup, the sampling frequency of the whole test bench is not known. Furthermore, the numerical discretization scheme, e.g., the Runge–Kutta method, as well as the implementation of interval operations in real-time environment is done with default settings of Simulink Embedded Coder. Hence, potential in order to speed up the algorithms or to increase the accuracy exist.

However, real-time capability has been shown up to a certain number of reduced-order interval observers. This may be improved with a detailed look into implementation as well as a real setup on which the bundle has to perform.

Additionally, with a detailed study about the interval observers within the bundle and the corresponding lookup tables with the parameters concerning the best enclosure for certain scenarios, interval observers with little improvement can be removed from the observer bundle which is then capable for practical use in real-time setup.

## 6. Conclusions

The design procedure for guaranteed state estimation of unmeasurable states is a challenging task. Hereby, the determination of an optimal observer gain depends on the chosen interval observer structure, the system properties, and the range of operation. The presented interval observer bundle’s underlying reduced-order hybrid interval observer does not restrict the configuration of the observer gain, but neither does it give specific statements about the resulting interval widths with respect to the eigenvalues nor the observer gain itself, especially not in case of parameter-varying systems. Therefore, a bundle of interval observers was proposed which overcomes the difficulty to choose an optimal observer gain by introducing a set of configurations covering the most important operating points with respect to the time-varying parameters.

The proposed methodical approach made huge improvements on the estimated interval widths of the induction machine’s magnetizing currents. The underlying reduced-order interval observers with their low computational effort yielded the possibility to supplement a bundle of interval observers for the given induction machine model with its small time constants. Furthermore, the offline optimization with its objective function’s decomposition and the adaptive gain give the potential to optimize the estimation for a priori considered operation areas, whereby, the continuity conditions are met.

Due to the continuity of the observer gain parameters, the switching of the configuration can not be done immediately. Therefore, the effect of the adaptive gains are delayed. Nevertheless, real-time capability as well as the verification of the proposed methods are successfully tested with measurement data of an induction machine and show a significant decrease of the estimated state’s interval width.

The applied number of interval observers within a bundle of observers as well as global optimization leading to a guaranteed and optimal enclosure of the estimated states should be considered for practical tasks.

Furthermore, the results within a real-time setup will be improved by applying instrumentation to measure the input voltages of the induction machine. Moreover, instrumentation with small measurement errors lead to an improvement of the interval widths for single observers and thus also for a bundle of observers.

A drawback of the proposed interval observer is the influence of the wrapping effect [33]. The over-approximation due to the interval calculations is amplified by the shape of the state-space representation, which also affects the calculation of the initial values at the time steps of re-initialization, which is attributed to the necessary transformation of the reduced-order observer.

Future work will be the consideration of uncertain parameters like the angular velocity $\omega_{L}\left(t\right)$. It is expected that the interval width can be kept within a feasible envelope for further applications like machine control or fault diagnosis.

## Figures and Tables

**Table 1:** Parameters of the induction machine under consideration.

$R_{r}$	$R_{s}$	$L_{h}$	$L_{\sigma s}$	$z_{p}$
12.6 m Ω	16.7 m Ω	1.3 m H	108.82 μ H	2

## Appendix A

With Equation (58), the matrix elements $h_{i,j}\left(t\right)$ of Equation (23) are given by:

(A1) $$\begin{array}{c} h_{11}\left(t\right)=-\frac{1}{L_{\sigma s}}\left(-\frac{L_{\sigma s}}{L_{h}}-\frac{R_{r}f_{11}L_{\sigma s}}{R_{r}^{2}+L_{h}^{2}\omega_{L}{\left(t\right)}^{2}}-\frac{f_{12}L_{h}\omega_{L}\left(t\right)L_{\sigma s}}{R_{r}^{2}+L_{h}^{2}\omega_{L}{\left(t\right)}^{2}}\right), \end{array}$$

(A2) $$\begin{array}{c} h_{12}\left(t\right)=-\frac{1}{L_{\sigma s}}\left(\frac{L_{h}L_{\sigma s}f_{11}\omega_{L}\left(t\right)}{R_{r}^{2}+L_{h}^{2}\omega_{L}{\left(t\right)}^{2}}-\frac{f_{12}L_{\sigma s}R_{r}}{R_{r}^{2}+L_{h}^{2}\omega_{L}{\left(t\right)}^{2}}\right), \end{array}$$

(A3) $$\begin{array}{c} h_{21}\left(t\right)=-\frac{1}{L_{\sigma s}}\left(-\frac{f_{21}L_{\sigma s}R_{r}}{R_{r}^{2}+L_{h}^{2}\omega_{L}{\left(t\right)}^{2}}-\frac{L_{h}L_{\sigma s}f_{22}\omega_{L}\left(t\right)}{R_{r}^{2}+L_{h}^{2}\omega_{L}{\left(t\right)}^{2}}\right), \end{array}$$

(A4) $$\begin{array}{c} h_{22}\left(t\right)=-\frac{1}{L_{\sigma s}}\left(-\frac{L_{\sigma s}}{L_{h}}-\frac{R_{r}f_{22}L_{\sigma s}}{R_{r}^{2}+L_{h}^{2}\omega_{L}{\left(t\right)}^{2}}+\frac{f_{21}L_{h}\omega_{L}\left(t\right)L_{\sigma s}}{R_{r}^{2}+L_{h}^{2}\omega_{L}{\left(t\right)}^{2}}\right). \end{array}$$

With Equation (58) the matrix elements $g_{i,j}\left(t\right)$ of Equation (24) are given by:

(A5) $$\begin{array}{cc} \; & g_{11}\left(t\right)=-\frac{1}{L_{h}{\left(L_{h}^{2}\omega_{L}{\left(t\right)}^{2}+R_{r}^{2}\right)}^{2}}L_{h}^{3}R_{r}\omega_{L}\left(t\right)(L_{\sigma s}(\omega_{L}\left(t\right)(f_{11}\left(f_{11}+f_{22}\right)\\ \; & -f_{12}\left(f_{12}-f_{21}+\omega_{L}\left(t\right)\right))+\omega_{L}\left(t\right)\left(f_{12}\left(f_{12}+\omega_{L}\left(t\right)\right)-f_{11}f_{22}\right)+2f_{11}{\dot{\omega }}_{L}\left(t\right))\\ \; & +f_{11}\omega_{L}\left(t\right)\left(R_{r}+R_{s}\right))+L_{h}^{2}R_{r}^{2}(L_{\sigma s}(f_{11}(\omega_{L}\left(t\right)\left(-f_{12}+f_{21}+\omega_{L}\left(t\right)\right)\\ \; & +\omega_{L}\left(t\right)\left(2f_{12}-f_{21}+\omega_{L}\left(t\right)\right))+f_{12}\left(f_{22}\omega_{L}\left(t\right)-{\dot{\omega }}_{L}\left(t\right)\right))+\omega_{L}\left(t\right)\\ \; & \;\cdot \;\left(R_{s}\left(f_{12}+2\omega_{L}\left(t\right)\right)+f_{12}R_{r}\right))+L_{h}R_{r}^{3}(L_{\sigma s}(f_{12}\left(f_{21}+\omega_{L}\left(t\right)-\omega_{L}\left(t\right)\right)\\ \; & +f_{11}^{2})+f_{11}\left(R_{r}+R_{s}\right))+L_{h}^{4}\omega_{L}{\left(t\right)}^{2}(\omega_{L}\left(t\right)\left(R_{s}\left(f_{12}+\omega_{L}\left(t\right)\right)+f_{12}R_{r}\right)\\ \; & +L_{\sigma s}\left(\omega_{L}\left(t\right)\left(f_{12}f_{22}+f_{11}\left(f_{12}+\omega_{L}\left(t\right)\right)\right)+f_{12}{\dot{\omega }}_{L}\left(t\right)\right))+R_{r}^{4}\left(f_{11}L_{\sigma s}+R_{s}\right)\\ \; & -{\dot{l}}_{f,11}\left(t\right), \end{array}$$

(A6) $$\begin{array}{cc} \; & g_{12}\left(t\right)=\frac{1}{L_{h}{\left(L_{h}^{2}\omega_{L}{\left(t\right)}^{2}+R_{r}^{2}\right)}^{2}}L_{h}^{2}R_{r}^{2}(L_{\sigma s}(\omega_{L}\left(t\right)\left(f_{12}^{2}-f_{12}\omega_{L}\left(t\right)-f_{11}f_{22}\right)\\ \; & +\omega_{L}\left(t\right)\left(f_{11}\left(f_{11}+f_{22}\right)-f_{12}\left(f_{12}-f_{21}+\omega_{L}\left(t\right)\right)\right)+f_{11}\left(-{\dot{\omega }}_{L}\left(t\right)\right))\\ \; & +f_{11}\omega_{L}\left(t\right)\left(R_{r}+R_{s}\right))+L_{h}^{4}\omega_{L}{\left(t\right)}^{2}(L_{\sigma s}(\omega_{L}\left(t\right)(f_{12}f_{21}-f_{12}\omega_{L}\left(t\right)\\ \; & +f_{11}^{2})+f_{11}{\dot{\omega }}_{L}\left(t\right))+f_{11}\omega_{L}\left(t\right)\left(R_{r}+R_{s}\right))\\ \; & -L_{h}^{3}R_{r}\omega_{L}\left(t\right)(L_{\sigma s}(f_{11}(\omega_{L}\left(t\right)\left(2f_{12}-f_{21}+\omega_{L}\left(t\right)\right)-\omega_{L}\left(t\right)(f_{12}-f_{21}\\ \; & +\omega_{L}\left(t\right)))+f_{12}\left(f_{22}\omega_{L}\left(t\right)+2{\dot{\omega }}_{L}\left(t\right)\right))+f_{12}\omega_{L}\left(t\right)\left(R_{r}+R_{s}\right))\\ \; & -L_{h}R_{r}^{3}\left(L_{\sigma s}\left(f_{12}f_{22}+f_{11}\left(f_{12}-\omega_{L}\left(t\right)+\omega_{L}\left(t\right)\right)\right)+f_{12}R_{r}+f_{12}R_{s}\right)\\ \; & -f_{12}L_{\sigma s}R_{r}^{4}-{\dot{l}}_{f,12}\left(t\right), \end{array}$$

(A7) $$\begin{array}{cc} \; & g_{21}\left(t\right)-\frac{1}{L_{h}{\left(L_{h}^{2}\omega_{L}{\left(t\right)}^{2}+R_{r}^{2}\right)}^{2}}L_{h}^{4}\omega_{L}{\left(t\right)}^{2}(L_{\sigma s}(\omega_{L}\left(t\right)(f_{21}\left(f_{12}+\omega_{L}\left(t\right)\right)\\ \; & +f_{22}^{2})+f_{22}{\dot{\omega }}_{L}\left(t\right))+f_{22}\omega_{L}\left(t\right)\left(R_{r}+R_{s}\right))+L_{h}^{3}R_{r}\omega_{L}\left(t\right)(L_{\sigma s}(f_{22}(\omega_{L}\left(t\right)\\ \; & \;\cdot \;\left(f_{12}-f_{21}+\omega_{L}\left(t\right)\right)-\omega_{L}\left(t\right)\left(f_{12}-2f_{21}+\omega_{L}\left(t\right)\right))+f_{21}f_{11}\omega_{L}\left(t\right)\\ \; & +2f_{21}{\dot{\omega }}_{L}\left(t\right))+f_{21}\omega_{L}\left(t\right)\left(R_{r}+R_{s}\right))+L_{h}^{2}R_{r}^{2}(L_{\sigma s}(\omega_{L}\left(t\right)(f_{21}(f_{12}-f_{21}\\ \; & +\omega_{L}\left(t\right))+f_{22}\left(f_{11}+f_{22}\right))+\omega_{L}\left(t\right)\left(f_{21}\left(f_{21}+\omega_{L}\left(t\right)\right)-f_{11}f_{22}\right)-f_{22}{\dot{\omega }}_{L}\left(t\right))\\ \; & +f_{22}\omega_{L}\left(t\right)\left(R_{r}+R_{s}\right))+L_{h}R_{r}^{3}(L_{\sigma s}\left(f_{21}f_{11}+f_{22}\left(f_{21}+\omega_{L}\left(t\right)\right)\right)\\ \; & +f_{21}R_{r}+f_{21}R_{s})+f_{21}L_{\sigma s}R_{r}^{4}\\ \; & -{\dot{l}}_{f,21}\left(t\right), \end{array}$$

(A8) $$\begin{array}{cc} \; & g_{22}\left(t\right)\frac{1}{L_{h}{\left(L_{h}^{2}\omega_{L}{\left(t\right)}^{2}+R_{r}^{2}\right)}^{2}}-L_{h}^{3}R_{r}\omega_{L}\left(t\right)(L_{\sigma s}(\omega_{L}\left(t\right)(f_{21}(f_{12}-f_{21}\\ \; & +\omega_{L}\left(t\right))+f_{22}\left(f_{11}+f_{22}\right))+\omega_{L}\left(t\right)\left(f_{21}^{2}-f_{21}\omega_{L}\left(t\right)-f_{11}f_{22}\right)+2f_{22}{\dot{\omega }}_{L}\left(t\right))\\ \; & +f_{22}\omega_{L}\left(t\right)\left(R_{r}+R_{s}\right))+L_{h}^{2}R_{r}^{2}(L_{\sigma s}(f_{22}(\omega_{L}\left(t\right)\left(f_{12}-f_{21}-\omega_{L}\left(t\right)\right)\\ \; & -\omega_{L}\left(t\right)\left(f_{12}-2f_{21}+\omega_{L}\left(t\right)\right))+f_{21}\left(f_{11}\omega_{L}\left(t\right)-{\dot{\omega }}_{L}\left(t\right)\right))+\omega_{L}\left(t\right)\\ \; & \;\cdot \;\left(R_{s}\left(f_{21}-2\omega_{L}\left(t\right)\right)+f_{21}R_{r}\right))-L_{h}R_{r}^{3}(L_{\sigma s}(f_{21}\left(f_{12}-\omega_{L}\left(t\right)+\omega_{L}\left(t\right)\right)\\ \; & +f_{22}^{2})+f_{22}\left(R_{r}+R_{s}\right))+L_{h}^{4}\omega_{L}{\left(t\right)}^{2}(\omega_{L}\left(t\right)\left(R_{s}\left(f_{21}-\omega_{L}\left(t\right)\right)+f_{21}R_{r}\right)\\ \; & +L_{\sigma s}\left(\omega_{L}\left(t\right)\left(f_{21}f_{11}+f_{22}\left(f_{21}-\omega_{L}\left(t\right)\right)\right)+f_{21}{\dot{\omega }}_{L}\left(t\right)\right))-R_{r}^{4}\left(f_{22}L_{\sigma s}+R_{s}\right)\\ \; & -{\dot{l}}_{f,22}\left(t\right). \end{array}$$

Whereby the elements ${\dot{l}}_{f,ij}\left(t\right)$ of Equation (63) are given by:

(A9) $$\begin{array}{cc} \; & {\dot{l}}_{f,11}\left(t\right)=\frac{L_{\sigma s}}{{\left(\omega_{L}{\left(t\right)}^{2}L_{h}^{2}+R_{r}^{2}\right)}^{2}}(2L_{h}^{2}\omega_{L}\left(t\right)\left(f_{12}\omega_{L}\left(t\right)L_{h}+f_{11}\left(t\right)R_{r}\right){\dot{\omega }}_{L}\left(t\right)\\ \; & -\left(\omega_{L}{\left(t\right)}^{2}L_{h}^{2}+R_{r}^{2}\right)\left(\omega_{L}\left(t\right){\dot{f}}_{12}\left(t\right)+f_{12}{\dot{\omega }}_{L}\left(t\right)\right)L_{h}+{\dot{f}}_{11}\left(t\right)R_{r}) \end{array}$$

(A10) $$\begin{array}{cc} \; & {\dot{l}}_{f,12}\left(t\right)=\frac{L_{\sigma s}}{{\left(\omega_{L}{\left(t\right)}^{2}L_{h}^{2}+R_{r}^{2}\right)}^{2}}(2\omega_{L}\left(t\right){\dot{\omega }}_{L}\left(t\right)\left(f_{12}R_{r}-L_{h}f_{11}\left(t\right)\omega_{L}\left(t\right)\right)L_{h}^{2}\\ \; & +\left(L_{h}\left(\omega_{L}\left(t\right){\dot{f}}_{11}\left(t\right)+f_{11}\left(t\right){\dot{\omega }}_{L}\left(t\right)\right)-R_{r}{\dot{f}}_{12}\left(t\right)\right)\left(\omega_{L}{\left(t\right)}^{2}L_{h}^{2}+R_{r}^{2}\right)) \end{array}$$

(A11) $$\begin{array}{cc} \; & {\dot{l}}_{f,21}\left(t\right)=\frac{L_{\sigma s}}{{\left(\omega_{L}{\left(t\right)}^{2}L_{h}^{2}+R_{r}^{2}\right)}^{2}}(2L_{h}^{2}\omega_{L}\left(t\right)\left(\left(f_{22}\left(t\right)\right)\omega_{L}\left(t\right)L_{h}+f_{21}\left(t\right)R_{r}\right)\\ \; & \;\cdot \;{\dot{\omega }}_{L}\left(t\right)-\left(\omega_{L}{\left(t\right)}^{2}L_{h}^{2}+R_{r}^{2}\right)\left(\omega_{L}\left(t\right){\dot{f}}_{22}\left(t\right)+\left(f_{22}\left(t\right)\right){\dot{\omega }}_{L}\left(t\right)\right)L_{h}+{\dot{f}}_{21}\left(t\right)R_{r}) \end{array}$$

(A12) $$\begin{array}{cc} \; & {\dot{l}}_{f,22}\left(t\right)=\frac{L_{\sigma s}}{{\left(\omega_{L}{\left(t\right)}^{2}L_{h}^{2}+R_{r}^{2}\right)}^{2}}(2\omega_{L}\left(t\right){\dot{\omega }}_{L}\left(t\right)\left(R_{r}\left(f_{22}\left(t\right)\right)-f_{21}\left(t\right)L_{h}\omega_{L}\left(t\right)\right)\\ \; & \;\cdot \;L_{h}^{2}+\left(L_{h}\left(\omega_{L}\left(t\right){\dot{f}}_{21}\left(t\right)+f_{21}\left(t\right){\dot{\omega }}_{L}\left(t\right)\right)-R_{r}{\dot{f}}_{22}\left(t\right)\right)\left(\omega_{L}{\left(t\right)}^{2}L_{h}^{2}+R_{r}^{2}\right)). \end{array}$$

## References

[1] Krebs S., Pfeifer M., Fugel S., Weigold J., Hohmann S. Interval observer for LPV systems based on time-variant transformations. Proceedings of the 55th IEEE Conference on Decision and Control.

[2] Huang J., Tan H. (2016). Development and Validation of an Automated Steering Control System for Bus Revenue Service. IEEE Trans. Autom. Sci. Eng..

[3] Fan W., Zhou Q., Li J., Zhu Z. (2017). A Wavelet-Based Statistical Approach for Monitoring and Diagnosis of Compound Faults with Application to Rolling Bearings. IEEE Trans. Autom. Sci. Eng..

[4] Shu S., Lin F. (2014). Fault-Tolerant Control for Safety of Discrete-Event Systems. IEEE Trans. Autom. Sci. Eng..

[5] Meskin N., Khorasani K. (2011). Fault Detection and Isolation.

[6] Wolff F., Krutina P., Krebs V. (2008). Robust Consistency-Based Diagnosis of Nonlinear Systems by Set Observation. IFAC World Congr..

[7] Raissi T., Puig V., Efimov D. (2020). Special issue on interval estimation applied to diagnosis and control of uncertain systems. Int. J. Control.

[8] Ding S.X. (2008). Model-Based Fault Diagnosis Techniques.

[9] Sun L., Alkhatib H., Kargoll B., Kreinovich V., Neumann I. (2019). Ellipsoidal and Gaussian Kalman Filter Model for Discrete-Time Nonlinear Systems.

[10] Krebs S., Schnurr C., Pfeifer M., Weigold J., Hohmann S. (2016). Reduced-order Hybrid Interval Observer for Verified State Estimation of an Induction Machine. Control Eng. Pract..

[11] Durieu C., Loron L., Sedda E., Zein I. Fault Detection of an Induction Motor by Set-Membership Filtering and Kalman Filtering. Proceedings of the European Control Conference.

[12] Kieffer M., Walter E. (2004). Guaranteed nonlinear state estimator for cooperative systems. Numer. Alg..

[13] Jaulin L., Kieffer M., Didrit O., Walter E. (2001). Applied Interval Analysis.

[14] Zbranek P., Vesely L. Nonlinear state estimation using interval computation in PMSM state observer simulation. Proceedings of the International Conference on Autonomous and Intelligent Systems.

[15] Krebs S., Gellrich T., Hohmann S. (2017). Interval observers for LPV systems and application to the guaranteed state estimation of an induction machine. IFAC World Congr..

[16] Meslem N., Ramdani N. (2011). Interval observer design based on nonlinear hybridization and practical stability analysis. Int. J. Adapt. Control Signal Process..

[17] Ramdani N., Meslem N., Candau Y. (2008). Reachability of Uncertain Nonlinear Systems Using a Nonlinear Hybridization. Hybrid Syst. Comput. Control.

[18] Müller M. (1926). Über das Fundamentaltheorem in der Theorie der gewöhnlichen Differentialgleichungen. Math. Z..

[19] Smith H.L. (1995). Monotone Dynamical Systems. Am. Math. Soc..

[20] Moisan M., Bernard O. (2005). Interval observers for non monotone systems application to bioprocess models. IFAC World Congr..

[21] Bernard O., Gouzé J.L. (2004). Closed loop observers bundle for uncertain biotechnological models. J. Process Control.

[22] Mairet F., Bernard O. Coupling framers to get enhanced interval observers. Application to growth rate estimation in a photobioreactor. Proceedings of the 48th IEEE Conference on Decision and Control.

[23] Moisan M., Bernard O., Gouzé J. (2009). Near optimal interval observers bundle for uncertain bioreactors. Automatica.

[24] Xu F., Puig V., Ocampo-Martinez C., Stoican F., Olaru S. (2014). Improved Fault Detection and Isolation Strategy using a Bank of Interval Observers. IFAC World Congr..

[25] Rotondo D., Efimov D., Cristofaro A., Johansen T.A. (2020). Estimation in uncertain switched systems using a bank of interval observers: Local vs glocal approach. IFAC Wrold Congr..

[26] Krebs S., Fugel S., Hohmann S. Interval State Observer Based on a Time-Variant Transformation for LPV Systems and Application to Induction Machines. Proceedings of the IEEE Conference on Control Technology and Applications.

[27] Krebs S., Bächle M., Hohmann S. (2018). Coupled boundary interval observer for LPV systems subject to uncertainties in input, output and parameters. Automatica.

[28] Angeli D., Sontag E.D. (2003). Monotone Control Systems. IEEE Trans. Autom. Control.

[29] Cellier F.E., Kofman E. (2006). Continuous System Simulation.

[30] Li Z., Zhang G., Diao L., Liu Z. Extended Kalman Filter based on inverse gamma model of induction motor. Proceedings of the Vehicle Power and Propulsion Conference.

[31] Quang N.P., Dittrich J. (2015). Vector Control of Three-Phase AC Machines: System Development in the Practice.

[32] Karmakar S., Chattopadhyah S., Mitra M., Sengupta S. (2016). Induction Motor Fault Diagnosis.

[33] Neher M., Jackson K.R., Nedialkov N.S. (2007). On Taylor Model Based Integration of ODEs. SIAM J. Numer. Anal..

[34] Nocedal J., Wright S. (2006). Numerical Optimization.

[35] Krebs S., Köhrer L., Hohmann S. Interval modelling of a voltage source inverter. Proceedings of the 8th International Conference on Power Electronics, Machines and Drives (PEMD 2016).

